# Natural stable isotope ratios and fatty acid profiles of estuarine tidal flat nematodes reveal very limited niche overlap among co-occurring species

**DOI:** 10.7717/peerj.7864

**Published:** 2019-10-11

**Authors:** Xiuqin Wu, Tania Campinas Bezerra, Dirk Van Gansbeke, Tom Moens

**Affiliations:** Biology Department, Marine Biology Lab, Ghent University, Gent, Belgium

**Keywords:** Stable isotopes, Fatty acids, Tidal flat, Biomarkers, Herbivory, Trophic niche, Marine nematodes, Predation, Omnivory

## Abstract

The high local-scale species diversity of marine meiofauna, and of nematodes in particular, has puzzled ecologists for decades. Both pronounced niche differentiation and neutral dynamics have been suggested as mechanisms underlying that high diversity. Differential resource use is the most plausible basis for niche differentiation, yet the vast majority of studies demonstrating that this is prominent in marine nematodes are based on laboratory experiments on single species or highly simplified assemblages. Only a small number of studies have investigated resource differentiation under natural conditions. Here we use natural stable-isotope ratios of carbon and nitrogen, as well as fatty-acid profiles, to assess differential resource use and trophic structure in nine abundant estuarine tidal flat nematode species, comprising different presumed feeding modes (deposit feeders, epistratum feeders, predators) and resource guilds (herbivores, carnivores) based on buccal cavity morphology. Nematodes comprise up to three different trophic levels (from primary to tertiary consumers), yet with the exception of some herbivores, omnivory is prominent. Bivariate isotopic niche spaces were of similar size among most species, irrespective of their trophic level. Herbivory not only contributed importantly to the nutrition of suspected herbivores, but also to that of species that were previously considered carnivores based on the morphology of their buccal cavity. Herbivory mainly targets diatoms in some nematode species, yet includes dinoflagellates in others. Bacteria, in contrast, appear to be of limited nutritional importance. *Odontophora setosus* is identified as a predator/omnivore (possibly of heterotrophic protists) with a trophic level in between that of secondary and tertiary consumers. Our study thus demonstrates that resource differentiation is pronounced among as well as within nematode feeding modes and resource guilds. However, this study included only the most abundant species of the in situ community, hence it remains to be established whether and to what extent its conclusions can be extrapolated to entire, often highly species-rich communities.

## Introduction

Estuarine tidal flat sediments are highly productive ecosystems, the productivity of which can be driven by a broad range of organic matter inputs, including deposited phytoplankton and particulate detritus of both terrestrial and marine origin, as well as of macroalgae, seagrasses, mangrove and/or salt marsh vegetation (Heip et al., 1995; Herman, Middelburg & Heip, 2001; Middelburg et al., 1996; Riera & Hubas, 2003; Riera, 2007). In most cases, however, the *in situ* productivity of microbial biofilms, i.e., complex consortia of benthic microalgae and heterotrophs embedded in a biogenic polymer matrix (Decho, 1990; Stal, 2010), fuels a major part of the secondary production on estuarine intertidal flats (Heip et al., 1995; Herman et al., 1999), and thus forms an important basis of estuarine food webs. Benthic biofilms play a pivotal role in carbon and nitrogen fluxes across the sediment-water interface (Hochard et al., 2010) and stabilize tidal flat sediment surfaces, thus reducing erosion (Paterson & Black, 1999; Stal, 2010). Nevertheless, several unknowns still exist about the complex interplay between microphytobenthos (MPB), benthic consumers and sediment properties.

Several studies have provided compelling evidence that MPB is the main basal resource fueling both a part of the macro- (Herman et al., 1999; Herman, Middelburg & Heip, 2001; Lebreton et al., 2011) and the majority of the meiofauna (mainly nematodes and harpacticoid copepods) (Carman & Fry, 2002; Cnudde et al., 2015; Moens, Bouillon & Gallucci, 2005; Moens et al., 2002; Moens et al., 2014; Rzeznik-Orignac et al., 2008) on estuarine intertidal flats. Nevertheless, there are typically at least some species which appear influenced by deposited phytoplankton or detritus, more so when tidal flats are more sheltered or have features that enhance deposition of suspended particulate organic matter, such as the presence of vegetation (Cnudde et al., 2015; Moens, Bouillon & Gallucci, 2005).

The high abundances and generally high biomass turnover rates of nematodes (Moens et al., 2013; Vranken & Heip, 1986) have caused many speculations about their importance in tidal flat sediments. The ecological importance of nematodes to soft-bottom marine ecosystems can be manifold (Schratzberger & Ingels, 2018): they can microbioturbate sediments, thereby influencing fluxes of oxygen and nutrients and affecting organic matter decomposition and biogeochemical cycles (Aller & Aller, 1992; Bonaglia et al., 2014; Cullen, 1973; Nascimento, Näslund & Elmgren, 2012). Their grazing and non-trophic interactions may affect the activity and community structure of both MPB and of sediment bacteria (D’Hondt et al., 2018; De Mesel et al., 2003; De Mesel et al., 2004) and thus probably also some of the ecosystem processes mediated by these micro-organisms (Hubas et al., 2010). Meiofaunal grazing rates may on average amount to 1% of MPB and bacterial biomass per hour, but a large variation around that average has been reported (Montagna, 1995). Indeed, nematodes consumed less than 1% of MPB production over a period of a few days in an in situ experiment in the Schelde estuary (the Netherlands) (Middelburg et al., 2000), while short-term (hours) meiofaunal consumption even exceeded MPB production in sediment slurries from San-Antonio Bay (USA) (Montagna & Yoon, 1991). Finally, benthic meiofauna can be an important food source for higher trophic levels, not only quantitatively, but also qualitatively because of the presence of high amounts of polyunsaturated fatty acids (PUFA) (De Troch et al., 2012; Leduc et al., 2015; Leduc & Probert, 2009), thus forming a potentially important link between primary producers and higher trophic levels (Coull, 1999; Danovaro et al., 2007).

However, many uncertainties remain about both qualitative and quantitative roles of nematodes in benthic ecosystem processes. This is often a consequence of the paucity of accurate information on nematode feeding ecology and feeding rates. While it is generally accepted that at the higher-taxon level, marine nematodes can consume a broad array of resources, including prokaryotes, auto-, mixo- and heterotrophic protists, and various benthic invertebrates (Jensen, 1987; Moens & Vincx, 1997; Moens, Yeates & De Ley, 2004), information on feeding ecology and resource partitioning at the species level remains very scant (Moens, Yeates & De Ley, 2004). As an example, while MPB is undoubtedly a pivotal carbon source for many intertidal nematodes (Moens, Bouillon & Gallucci, 2005; Rzeznik-Orignac et al., 2008), the pathways from MPB to nematodes are not always very clear. For example, there is debate whether nematode species obtain the MPB carbon directly through herbivory or indirectly, for instance through bacteria and/or herbivorous protists that feed on MPB and its extracellular polymeric substances (EPS) (Decho, 2000; Moens, Bouillon & Gallucci, 2005; Moens et al., 2014).

There is a widespread habit to assign marine nematodes to a limited number of feeding types, largely based on the morphology of the feeding apparatus (Wieser, 1953; Jensen, 1987; Moens & Vincx, 1997). Not only do such feeding-type classifications funnel the high species diversity of marine nematodes into a very limited trophic diversity, they also act as black boxes, ignoring the flexibility that nematodes may exhibit depending on food availability and/or competitive interactions with other benthic invertebrates (Moens & Vincx, 1997; Moens, Yeates & De Ley, 2004). Equally problematic from an ecosystem functioning point of view, is that the feeding guilds largely reflect feeding mode rather than resources (Moens, Yeates & De Ley, 2004). For example, both deposit feeders and epistratum feeders probably graze on (the same?) benthic microalgae, but in different ways. Predators/omnivores are capable of predation on other benthic invertebrates and/or heterotrophic protists (Hamels et al., 2001; Moens & Vincx, 1997), but at least some of these species may be very flexible feeders that can switch to herbivory (Franco et al., 2008; Moens et al., 2014) or bacterivory (Moens, Verbeeck & Vincx, 1999), depending on resource availability. A direct consequence of our lack of species-level knowledge on nematode feeding ecology, is that the role of resource selectivity as a driver of the often species-rich local assemblages remains a matter of debate (Moens & Beninger, 2018). Indeed, although it has been suggested that most marine nematodes may be flexible feeders (Moens, Yeates & De Ley, 2004), it is unclear to what extent species within and among feeding groups compete for resources. It is equally unclear whether those that utilize MPB as a resource, do so selectively or rather non-selectively.

A combination of dual stable isotope and fatty acid profiles has proven useful in examining food-web interactions and in tracing an animal’s diet (Neubauer & Jensen, 2015). Natural stable isotope ratios of carbon and nitrogen can provide good pointers to the basal resources fuelling food webs, as well as to the trophic level of consumers (Peterson & Fry, 1987; Post, 2002; Vander Zanden & Rasmussen, 2001). On the other hand, this technique has a limited resolution with respect to identifying which primary producers act as a basal resource, because different primary producers in tidal flat biofilms often have no or only very limited isotopic differences (Mutchler, Sullivan & Fry, 2004). Fatty acid (FA) profiles of consumers and their resources may offer complementary information that can allow to further disentangle food-web links (Neubauer & Jensen, 2015), for instance, because certain primary producers (e.g., diatoms and dinoflagellates) with overlapping stable-isotope signatures have distinct FA biomarkers. Combined use of stable isotopes and FA in marine nematodes has nevertheless remained rare (but see Braeckman et al., 2015; Guilini et al., 2013; Leduc, 2009; Leduc et al., 2015; Leduc & Probert, 2009; Van Campenhout & Vanreusel, 2016; Van Gaever et al., 2009), mainly because of the large numbers of specimens that need to be pooled to obtain reproducible measurements, and of the difficulty in processing such numbers with a reasonable taxonomic or functional resolution.

Against the background of several published papers which have convincingly demonstrated that nematodes on estuarine tidal flats are largely fuelled by MPB carbon (Carman & Fry, 2002; Moens, Bouillon & Gallucci, 2005; Moens et al., 2002; Moens et al., 2014; Rzeznik-Orignac et al., 2008), the main aim of the present paper was not to demonstrate the contribution of different resources of tidal-flat nematodes, but to disentangle the trophic structure of the ‘nematode food web’ on these tidal flats. Specifically, we determined natural stable carbon and nitrogen isotopes as well as fatty acid profiles of nine abundant nematode species, representing different feeding guilds. First, we assessed the trophic level of several nematode species which are presumed to be mainly consumers of MPB and of others which are known as predators, and evaluated the hypothesis that these represent clearly distinct trophic levels, i.e., primary and secondary consumers, respectively. Secondly, our sampling comprised multiple species each that under the previous hypothesis would classify as primary and secondary consumers, allowing us to test the degree of resource partitioning among species of presumed similar trophic level. We used isotopic niche spaces as well as multivariate analysis of fatty acid profiles to assess this concept. Thirdly, we used fatty acid biomarkers to investigate the contribution, if any, of hitherto poorly documented resources such as dinoflagellates and zooplankton (dead and/or faecal pellets) in the diet of intertidal nematodes. In addition to these main aims, we also assessed the following specific hypotheses: (a) microalgal grazers which ingest their prey whole are more likely to co-ingest bacteria and EPS, and will therefore have higher contributions of bacterial biomarkers in their diet; (b) omnivory is common in nematodes with presumed predatory ecology (Moens, Yeates & De Ley, 2004).

## Materials and Methods

### Sampling site, sampling procedure and collection of nematodes

Sampling was conducted at the Paulina intertidal flat (Cnudde et al., 2015; Gallucci, Steyaert & Moens, 2005) in the polyhaline reach of the Schelde Estuary, SW Netherlands. This tidal flat is characterised by a high heterogeneity in sediment types, which range from silty in the more downstream parts to medium sandy at the most upstream portion of the tidal flat. Moreover, there is a salt marsh bordered by muddy sediments in the downstream part of this intertidal area.

**Figure 1:**
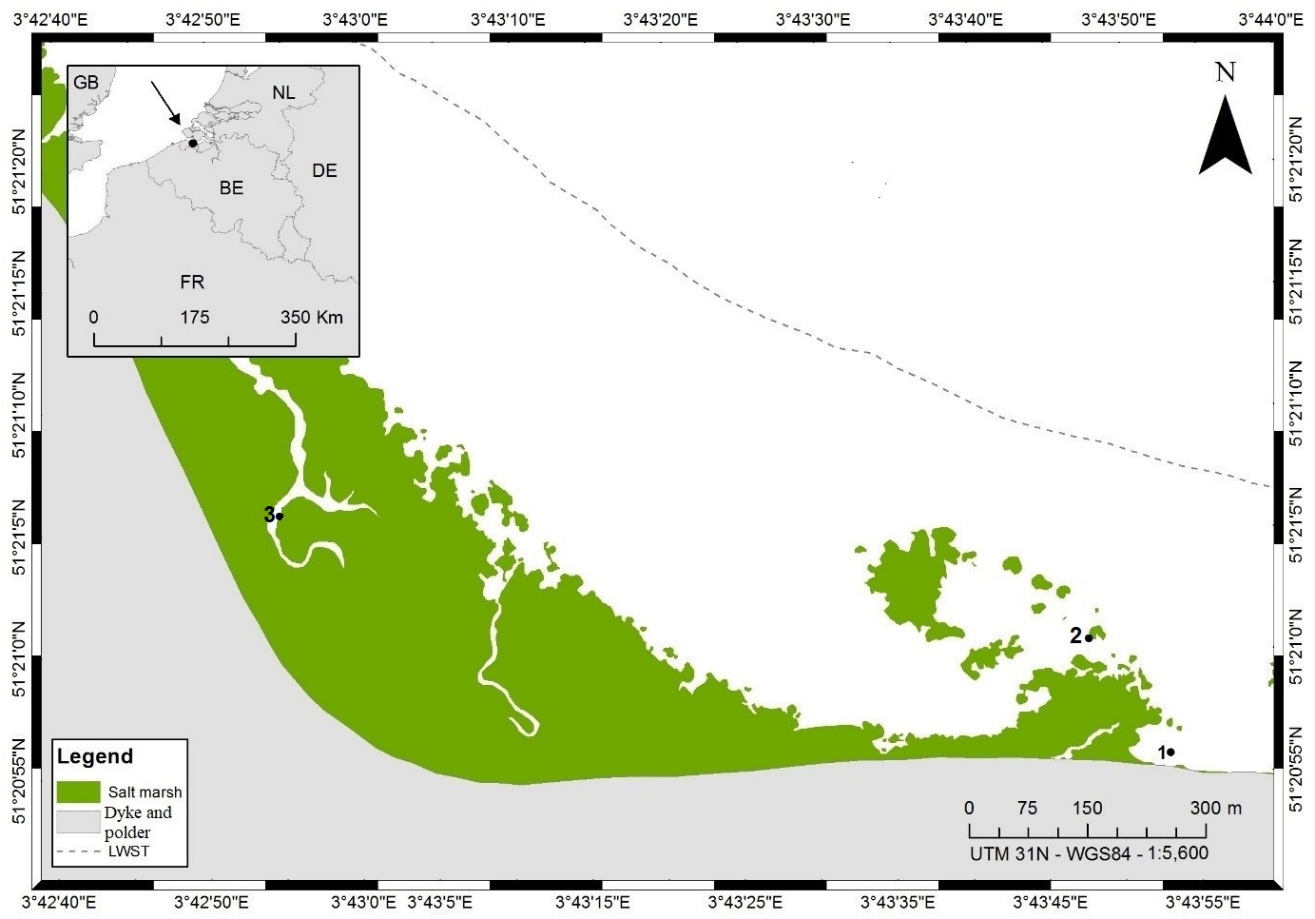
Map of sampling locations at the Paulina polder intertidal flat, The Netherlands. Numbers indicate the different sampling stations, LWST indicates the mean low water spring tide and the mean high water spring tide coincides with the dyke. This figure was created by Renata Mamede da Silva Alves.

Our samples for stable isotope analyses were collected in a transition zone with a dynamic mosaic of patches of different sediment compositions (Cnudde et al., 2015; Gallucci, Steyaert & Moens, 2005) in an area of ca. 200 × 200 m (stations 1 and 2, Fig. 1). Whereas the nematode communities inhabiting the extremes of the sedimentary gradient from muddy to sandy are very different (Wu et al., 2019; Gallucci, Steyaert & Moens, 2005), within the transition zone, patches which differ more subtly in granulometry have different yet partly overlapping community compositions. Based on prior knowledge of the area (Wu et al., 2019; Bezerra & Moens, 2012, unpublished data), we *a priori* identified 8 genera (Table 1) that are typically abundant in fine- to medium-sandy sediments with a relatively low silt content (≤15%) at this tidal flat, such as in stations 1 and 2. Nematode abundance of these two stations in the top 6 cm is very similar (523 ± 132 and 580 ± 120 individuals.10 cm^−2^ in stations 1 and 2, respectively), whereas genus diversity is a little higher in st1 than in st2: the expected number of genera in a sample of 100 nematodes (EG(100)) was 25.9 ± 2.64 and 20.7 ± 2.66, respectively, whereas Shannon–Wiener diversity was 2.99 ± 0.14 in st1 and 2.54 ± 0.21 in st2 (Wu et al., 2019). A ninth genus (Table 1) that only occurred in silty sediment (station 3, Fig. 1) was included here because of its hitherto completely unresolved feeding ecology. We sampled two sites (st1, st2) in the above-mentioned transitional area and an additional one in a silty gully of the salt marsh (st3), where some of our target species also reach high abundances. Samples for stable isotope analysis (SIA) were collected from the two sites in the transitional area only, except for the ninth nematode species, *Odontophora setosus* (see below), whereas samples for fatty acid analysis (FAA) originated from either the transitional area or the salt marsh gully or both. Samples for SIA and FAA were collected in the same season (late spring, June) but in different years: 2010 for the SI samples and 2014 for the FA samples.

Sediment samples for the extraction of nematodes were collected in a non-quantitative way by scraping the top 1–2 cm of sediment off using a small shovel and pooling it per site into a bucket. The collected sediment was hand-mixed in the field and—upon return to the lab—incubated overnight at environmental temperature with a thin layer of habitat water on top. During this incubation, many nematodes move from deeper layers towards the surface, hence even fairly small subsamples from the surface layer in the buckets tend to yield high abundances of live nematodes. Nematodes were extracted alive by simple, repeated decantation over a 63-µm (for the more slender species of *Odontophora, Theristus*) or a 125-µm (for all other species) mesh size sieve after vigorous stirring of samples with a jet of tap water. This procedure facilitates release of the nematodes from the sediments (Somerfield, Warwick & Moens, 2005) and was repeated 5 to 10 times. This mesh size of the sieves was chosen to reduce the retention of fine particulate matter on the sieves, and to enhance the proportion of adult specimens, which we preferentially selected for our analyses. The nematodes were then harvested from the sieve using a small volume of sterile artificial seawater (ASW (Dietrich & Kalle, 1957)) of ambient salinity and stored in the dark at 4 °C until further processing. We avoided other sample preservation methods such as freezing of samples, because certain fatty acids are unstable and could be partly broken down by thawing (Murphy, Mann & Sinclair, 2003).

**Table 1:** Size and feeding type of the nematode species and the number of specimens used for stable isotope and fatty acid analyses. Nematode body width and length and feeding type according to Moens & Vincx (1997). Numbers of replicate samples for stable isotope (SI) and fatty acids (FA) analysis, and number of specimens pooled per replicate sample (left and right of the comma: SI and FA analysis, respectively) are also indicated.

Species	Width (µm)	Length (µm)	Feeding type	Replicate number SI	Replicate numbers FA (st1, st2, st3)	Number of specimens forSI and FA
*Theristus acer*	44 ± 3	1,780 ± 67	DF	1	3,0,3	180, 200
*Daptonema hirsutus*	72 ± 17	1,640 ± 97	DF	3	0,0,4	100–170, 60
*Praeacanthonchus punctatus*	73 ± 8	1,822 ± 102	DF/EF	4	2,4,4	50-85,100
*Metachromadora remanei*	59 ± 12	1,275 ± 70	EF	6	3,4,0	120–150,160
*Enoploides longispiculosus*	118 ± 5	3,020 ± 194	P/FP	7	2,0,0	75–80, 30
*Adoncholaimus fuscus*	165 ± 17	4,934 ± 30	FP	7	3,0,0	35–40,15
*Oncholaimus oxyuris*	62 ± 2	3,800 ± 120	FP	1	3,0,0	80, 20
*Enoplus brevis*	176 ± 1	7,000 ± 800	P/FP	3	0,3,0	5–10, 21
*Odontophora setosus*	34 ± 1	3,050 ± 351	?	2	0,3,0	200, 190

**Notes.**

DF deposit feeder EF epigrowth feeder P predator FP facultative predator

The feeding guild of *Odontophora* is unknown.

### Selection of nematode taxa for stable-isotope and fatty-acid analyses

We chose a selection of locally abundant nematode genera (Table 1) that encompass a variety of traits, including different body sizes, feeding habits and—presumably—trophic levels.

*Theristus acer* is a deposit feeder that ingests diatoms, other microalgae and perhaps other unicellular organisms, particle size determining the upper limit of food items that can be ingested (Boucher, 1973; Moens & Vincx, 1997). *Daptonema hirsutum* belongs to the same family and feeding type, yet is considerably larger and wider than *T. acer*, and hence may be expected to be capable of ingesting a broader range of food particles. In both *Theristus* and *Daptonema*, diatom frustules are commonly observed in the gut, supporting their contribution to the diet of these nematodes (Moens & Vincx, 1997; Nehring, 1992).

*Praeacanthonchus punctatus* has been listed as an epistratum feeder because of its buccal armature (Wieser, 1953). However, observations indicate that it mostly swallows whole prey, much like the above-mentioned deposit feeders. Either way, this species actively grazes on diatoms (Moens et al., 2014). Herbivory has also been proposed as the main feeding strategy of *Metachromadora remanei* (Moens, Bouillon & Gallucci, 2005), although this genus was initially classified as a predator based on its strong tooth and muscular pharynx (Wieser, 1953). *Metachromadora remanei* does not ingest its food whole but pierces cells with its tooth, then sucks out their contents (Moens, Bouillon & Gallucci, 2005).

The four species mentioned thusfar are considered primary or secondary consumers (as bacterivory may also occur), although in a stable-isotope study on the feeding ecology of nematodes in a *Zostera* seagrass bed, the genera *Metachromadora* and *Daptonema* did not stand out as grazers of microphytobenthos or epiphytic microalgae, but rather of fungi and/or bacteria associated with decomposing *Zostera* detritus (Vafeiadou et al., 2014).

The remaining species are considered secondary or higher-order consumers. *Enoploides longispiculosus* was long considered a strict predator of other nematodes (Moens et al., 2000), ciliates (Hamels et al., 2001) and other small benthic invertebrates (Moens & Vincx, 1997), but it can also graze on microalgae (Franco et al., 2008; Moens et al., 2014). Oncholaimidae, such as *Adoncholaimus fuscus* and *Oncholaimus oxyuris*, are capable of predation on other nematodes, but probably have other feeding strategies as well, perhaps including bacterivory (Moens, Verbeeck & Vincx, 1999). Microalgae are only rarely seen in their intestines. They have been classified as facultative predators, where strategies other than predation are poorly understood, although they may encompass some form of deposit feeding (Meyers, Hopper & Cefalu, 1970). *Enoplus brevis* is a generalist feeder which is capable of ingesting a broad range of prey, from cyanobacteria over microalgae to many benthic invertebrates (Hellwig-Armonies, Armonies & Lorenzen, 1991). Finally, *Odontophora setosus* strongly resembles genera that are commonly believed to be deposit feeders. They are long and very slender nematodes with fairly narrow mouth openings, yet they do possess a buccal cavity with cuticularised walls and a ring of six odontia, which could point to a predatory feeding strategy; their assignment to a feeding type therefore remains dubious (Austen, Widdicombe & Villano-Pitacco, 1998). We only encountered this species in silty sediments in the salt marsh, but decided to include it as it is a common genus in many coastal nematode assemblages, and because empirical information on its feeding ecology is totally lacking. Henceforth, we refer to these nine species by their genus name.

### Preparation of nematode samples for stable-isotope and fatty-acid analyses

After decantation (see above, section ‘Sampling site, sampling procedure and collection of nematodes’), nematodes were maintained alive in sterile ASW with a salinity of 25 psu in the fridge until further sample processing. This sample processing was performed within 2 days after field sampling. Adult and fourth-stage juvenile nematodes (males and mostly non-gravid females) were picked alive one by one on the tip of a tungsten wire under a Leica M5 binocular and transferred to sterile ASW to rinse off adhering particles, then—in the case of nematodes collected for SIA—transferred again one by one to precombusted (4 h at 500 °C) 2.5 × 6 mm aluminium cups (Elemental Microanalysis Ltd) with a few drops of milliQ water. These cups were kept upright in a multiwell plate and allowed to dry for 3 h at 60 °C, after which they were pinched close with sterile forceps, and kept under dry atmosphere until isotopic analysis (Moens, Bouillon & Gallucci, 2005; Moens et al., 2014). No removal of lipids was performed. We used available data on mean individual biomass (calculated from nematode length and width (Somerfield, Warwick & Moens, 2005)) of the species used here at the location of sampling (Table 1) to estimate how many specimens had to be pooled per cup to ensure that enough biomass was available for reliable C and N analysis (≥5 µg of each element). Given the large differences in nematode size and biomass, this implies that very different numbers of specimens were pooled for different species (Table 1).

Nematodes for FA analysis were hand-sorted in much the same way as for SIA. However, instead of transferring them to aluminium cups, they were stored in 2.5-ml GC vials with ASW. Immediately after transfer of the last nematode, a vial was centrifuged for 6 min at 1,800 g, and the supernatant ASW replaced by milliQ water for rinsing during a final centrifugation step, after which most of the supernatant milliQ water was gently siphoned off and the pellet with the nematodes was immediately stored frozen at −80 °C and later freeze-dried.

Microphytobenthos was isolated from station 1 after migration through lens tissue onto plastic cover slips (Moens, Bouillon & Gallucci, 2005), which yielded clean, diatom-dominated biofilms. These were collected, concentrated and lyophilised. Aliquots containing >5 µg N were weighed into 5 × 8 mm silver cups (Elemental Microanalysis Ltd), acidified in situ with dilute HCl to remove carbonates, and dried, after which the cups were pinched close and stored under dry atmosphere until stable isotope analysis.

### Stable isotope analysis

The aluminium cups containing nematodes were combusted in a ThermoFinnigan 1112 elemental analyser coupled online through a Conflo III interface to a ThermoFinnigan Delta Plus XL isotope ratio mass spectrometer for the simultaneous analysis of C and N isotopes. Isotope ratios are expressed as *δ* values in units of ‰relative to the conventional standards, i.e., Vienna Peedee Belemnite for C and atmospheric N_2_ for N, *δ* being equal to (R_sample_/R_standard_ −1) × 1000 (Fry, 2006). In this formula, R is the ratio of the heavy to the light isotope. Analytical precision of both *δ*^13^C and *δ*^15^N measurements was ≤0.2‰. IAEA (International Atomic Energy Agency) standards CH6 (sucrose) and N1 (ammonium sulphate) were used as external standards, with at least one standard being measured after every 10 regular samples.

All *δ*^13^C values so obtained were corrected for possible carbon contamination of sample cups according to the procedure described in Moens et al. (2014). No such correction was required for *δ*^15^N data.

### Fatty acid analysis

The freeze-dried nematode samples were subjected to a slightly adapted (in terms of reagent volumes) version of the protocol by Masood, Stark & Salem (2005) to extract lipids and prepare fatty acid methyl esters (FAMEs). FAMEs were analyzed, identified and quantified following De Troch et al. (2012). In short, we performed gas chromatography-mass spectrometry in splitless mode with a Hewlett Packard 6890N gas chromatograph coupled to an HP 5973 mass spectrometer, using the same injection and running time parameters as De Troch et al. (2012). FAMEs so obtained were identified by comparing their retention times and mass spectra with those of authentic standards and available ion spectra in WILEY mass spectral libraries and analysed with the software MSD ChemStation (Agilent Technologies), using external standards (SupelcoTM 37 Component FAME Mix, Supelco # 47885, Sigma-Aldrich Inc., USA) for individual FAME quantification (De Troch et al., 2012). FA concentrations were determined by reference to the internal standard C19:0. Fatty acid notation is in the form of A:B *ω*X, where A represents the number of carbon atoms, B gives the number of double bonds and X is the position of the double bond closest to the terminal methyl group (Guckert et al., 1985).

### Fatty acid biomarkers

Although the usefulness of some fatty acid biomarkers depends on habitat and environmental conditions (Parrish et al., 2000), we applied biomarkers which have repeatedly been used in temperate estuarine environments (Kelly & Scheibling, 2012).

Diatoms, which usually form by far the main component of microphytobenthos on tidal flats in the polyhaline reach of the Schelde Estuary (Hamels et al., 1998; Sabbe & Vyverman, 1991), were indicated by the concentration of C16:1ω7 (Dalsgaard et al., 2003) as well as by the ratio of C16:1/C16:0 (Claustre et al., 1988). Longer-chain FA like eicosapentaenoic acid (EPA) are abundant in, but not unique to, diatoms. Docosohexaenoic acid (DHA) only occurs in limited abundance in diatoms, but is prominently present in dinoflagellates, which can also form an important part of MPB. Hence, we applied the ratio DHA/EPA as a measure of the relative importance of dinoflagellates *vs* diatoms, higher values indicating a higher prominence of dinoflagellates (Kelly & Scheibling, 2012; Parrish et al., 2000). When concentrations of C_18_PUFA (polyunsaturated fatty acids) are low (≤3%), the contributions of SFA (saturated fatty acids) (C14:0 + C16:0 + C18:0) can be used as indicators of feeding on dinoflagellates and prymnesiophytes (Braeckman et al., 2015; Dalsgaard et al., 2003)*.*

We used the sum of FA C15:0 and C17:0 to indicate feeding on prokaryotes in general (Kelly & Scheibling, 2012; Parrish et al., 2000), whereas C18:1ω7 has previously been used as a general bacterial marker and as a marker of chemoautotrophic bacteria (Cnudde et al., 2015; Van Gaever et al., 2009).

Other sources, such as salt marsh vascular plants and green algae, were indicated by C18:1ω9 (Kelly & Scheibling, 2012), whereas vascular plant detritus of terrestrial origin was indicated by a sum of LC-SFA (C20-C24) (Cnudde et al., 2015; Douglas et al., 1970). Microzooplankton was indicated by arachidonic acid (ARA, 20:4ω6) (Parrish et al., 1995), and zooplankton by a sum of C20:1 and C22:1 (Parrish et al., 2000). Finally, we used the ratio of PUFA/saturated FA (PUFA/SFA) and the abundance of 20:1ω9 as indicators of carnivory (Cripps & Atkinson, 2000).

### Data analysis

### Dual stable isotope data

We visually inspected dual (C + N) isotope plots as a first pointer to major carbon sources and to the trophic level of nematode taxa. Given the existence of previous studies highlighting the predominant contribution of MPB to nematodes at this (and other) tidal flat(s) (Moens, Bouillon & Gallucci, 2005; Moens et al., 2002; Moens et al., 2014), our goal was not to investigate in detail the contributions of different carbon sources to the diets of nematodes, but rather to reconstruct the nematode part of a benthic food chain from MPB to higher trophic levels and to assess resource divergence and overlap between different nematode taxa. We used the formula

\begin{eqnarray*}& & TL=({\delta }^{\text{15}}{N}_{\mathrm{consumer}}-{\delta }^{\text{15}}{N}_{\mathrm{baseline}})/FF+T{L}_{\mathrm{baseline}} \end{eqnarray*}

to estimate trophic level, where TL = trophic level, baseline is an organism of known trophic level, and FF is the N fractionation factor at trophic transfer (Post, 2002). Given the variability of the FF (McCutchan et al., 2003), we used two scenarios, one with the often proposed FF of 3.4 (Minagawa & Wada, 1984), the other with an FF value of 2.5‰ as proposed by Vander Zanden & Rasmussen (2001). This comparison allowed us to assess if, and to what extent different FF scenarios affect the main conclusions on nematode trophic level. Each of these two scenarios was run for two different baseline organisms: one with MPB as a primary producer at trophic level 1, the other with *Metachromadora remanei* as a herbivore at trophic level 2 (Moens, Bouillon & Gallucci, 2005). The latter was done because the present and a previous study found a large offset in *δ*^15^N (FF close to 5) between MPB and the nematodes with lowest *δ*^15^N (see Moens et al., 2014, for possible explanations). We have dual stable-isotope data from bimonthly samplings at Paulina in 2010, and these demonstrate that all-year long, *M. remanei* consistently had (one of) the lowest *δ*^15^N of all nematode species analysed. It is therefore plausible that this species is a first-order consumer which feeds primarily as a herbivore on MPB (Moens, Bouillon & Gallucci, 2005; Bezerra & Moens, 2012, unpublished data).

We further used our stable-isotope data to calculate two descriptive metrics that assess the niche width of consumers, i.e., convex hull volumes (CHV) (Layman et al., 2007) and standard ellipse areas (SEA) (Jackson et al., 2011). While CHV provide a suitable representation of niche width, they are rather sensitive to small sample sizes (Jackson et al., 2011), an issue which is less important for SEA, which use Bayesian inference and allow robust comparisons with data sets comprising different sample sizes. When sample size is low, as is the case in our study, a corrected SEA (SEAc) is calculated which leads to a slightly larger ellipse but with the same geometrical shape as SEA (Jackson et al., 2011). The SEAc, containing ∼40% (default value in SIBER) of the data (centred on the mean and SDs of the bivariate data as semi-axes), and convex hulls were used to delineate isotopic niche spaces per nematode species. Differences between species in these standard ellipse areas, as well as niche overlap among the ellipses of different species, were calculated using Bayesian inference based on 10,000 posterior probabilities drawn from the SEAc model. These isotope-based metrics were analysed in the SIBER package in R (Jackson et al., 2011).

### Fatty acid profiles and biomarker concentrations

All analyses were done in Primer (v6.0) with PERMANOVA add-on (Anderson, Gorley & Clarke, 2008).

We determined the total amount of FA (TFA) in our nematode samples, and identified different major FA classes based on the degree of their saturation: SFA (saturated FA), PUFA (polyunsatured FA), HUFA (highly unsaturated FA), MUFA (mono-unsaturated FA), as well as different PUFA classes based on the position of the first double bond relative to the terminal methyl group: ω3PUFA and ω6PUFA. Differences in the concentrations of these FA classes between nematode species were examined with one-way PERMANOVA’s based on a Euclidean distances matrix, in which all samples of a given species were considered replicates, irrespective of their station of origin.

Secondly, non-metric multidimensional scaling (nMDS) was used to visualize differences in the multivariate fatty acid composition of nematode species; we chose a Bray-Curtis similarity matrix on the basis of the relative fatty acid concentrations (Legendre & Legendre, 2012). Each individual nematode sample was plotted separately in the nMDS.

PERMANOVA was then used to formally identify statistically significant differences in the FA composition or in the concentrations of specific FA biomarkers or in biomarker ratios between nematode species. Firstly, a one-way PERMANOVA was performed on the whole dataset, to assess differences in FA composition/concentration/ratio between nematode species. In this analysis, all samples of a given genus were considered replicates, irrespective of their station of origin. To address the possibility of station differences within a nematode species, a two-way PERMANOVA was performed with factors species (three levels: *M. remanei, P. punctatus, T. acer*) and station (two levels: st1 and st3) on a dataset composed of all data of genera that were collected from more than one location. Pairwise tests were done on significant factor(s) or interaction terms. Because PERMANOVA is sensitive to heterogeneity of variances (dispersion effect), PERMDISP was used to test whether significant differences were due to treatment (location) or to variance effects.

SIMPER (Similarity Percentage Analysis) analysis was conducted to identify which fatty acids contributed most to the dissimilarity among species.

## Results

### Trophic level and resources of nematodes based on SIA

**Figure 2:**
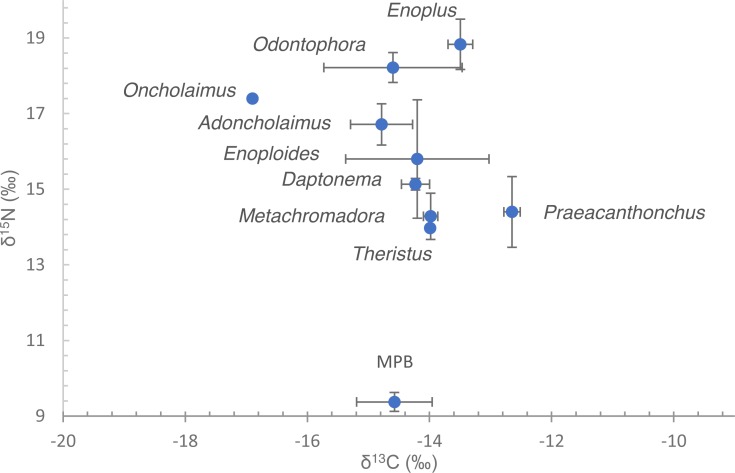
Dual stable isotope data (*δ*^13^C and *δ*^15^N) of nematodes and microphytobenthos. Data are means of the numbers of replicates listed in Table 1 with standard deviation. Nematode species are indicated by their genus name. Note that for* Oncholaimus* and *Theristus*, only a single measurement was available.

Nematode *δ*^13^C values exhibited a small range, from −12.6 ± 0.13 to −16.9‰ (Fig. 2, Table 2). Omission of *Oncholaimus* further reduced that range to −12.6 ± 0.13 to −14.8 ± 0.51‰. These values largely correspond to measured and previously published data on MPB on this intertidal area (Moens, Bouillon & Gallucci, 2005; Moens et al., 2002; Moens et al., 2014).

**Table 2:** Natural stable isotope signatures (*δ*^13^C and *δ*^15^N) and estimated trophic levels of nine nematode species from a temperate tidal flat. Nematode trophic level (TL) was calculated from the *δ*^15^N according to 4 scenarios: with a trophic-level fractionation of 3.4 (TLa) and one of 2.5‰(TLb), and for both fractionation factors, one with MPB as the reference trophic level (TL = 1) and one with *Metachromadora* as the reference level (TL = 2) (Moens, Bouillon & Gallucci, 2005). Trophic levels marked with * were assigned, not calculated.

Genus	*δ*^15^N	*δ*^13^C‰	TLa_MPB	TLb_MPB	TLa_M	TLb_M
*Enoplus*	18.83 ± 0.67	−13.49 ± 0.20	3.8	4.8	3.3	3.8
*Odontophora*	18.22 ± 0.40	−14.60 ± 1.13	3.6	4.5	3.2	3.6
*Oncholaimus*	17.4	−16.88	3.4	4.2	2.9	3.2
*Adoncholaimus*	16.71 ± 0.55	−14.79 ± 0.51	3.2	3.9	2.7	3.0
*Enoploides*	15.84 ± 0.36	−13.63 ± 0.36	2.9	3.6	2.5	2.6
*Daptonema*	15.13 ± 0.15	−14.23 ± 0.23	2.7	3.3	2.3	2.3
*Praeacanthonchus*	14.4 ± 0.94	−12.65 ± 0.13	2.5	3.0	2.0	2.0
*Metachromadora*	14.28 ± 0.61	−13.98 ± 0.12	2.4	3.0	2.0*	2.0*
*Theristus*	13.97	−13.99	2.4	2.8	1.9	1.9
MPB	9.38 ± 0.25	−14.58 ± 0.62	1.0*	1.0*		

*δ*^15^N of nematodes spanned a range between 14.0‰in *Theristus* and 18.9 ± 0.67‰in *Enoplus* (Fig. 2, Table 2). Depending on the trophic fractionation factor and trophic baseline used, nematodes occupied trophic levels from 2 up to almost 5 (Table 2). Specifically, when using MPB as a baseline (TL = 1) and a FF of 3.4‰, trophic level varied between 2.4–2.5 for *Theristus, Metachromadora* and *Praeacanthonchus* and 3.8 for *Enoplus*, with a majority of species clustering at TL’s between 2.7 and 3.4. Still with MPB as a baseline but with a FF of 2.5‰, this range expanded from a TL close to 3 for *Theristus, Metachromadora* and *Praeacanthonchus* to values in excess of 4.5 for *Enoplus* and *Odontophora*, with a majority of species having a TL of 3.3–4 (Table 2).

When using *Metachromadora* as a baseline and a FF of 3.4‰, nematode TL ranged from close to 2 for *Theristus* and *Praeacanthonchus* to in between 3 and 3.5 for *Enoplus* and *Odontophora*. With a FF of 2.5, the corresponding TL’s remained unaltered for *Theristus* and *Praeacanthonchus*, but increased to values in between 3.5 and 4 for *Enoplus* and *Odontophora* (Table 2).

**Figure 3:**
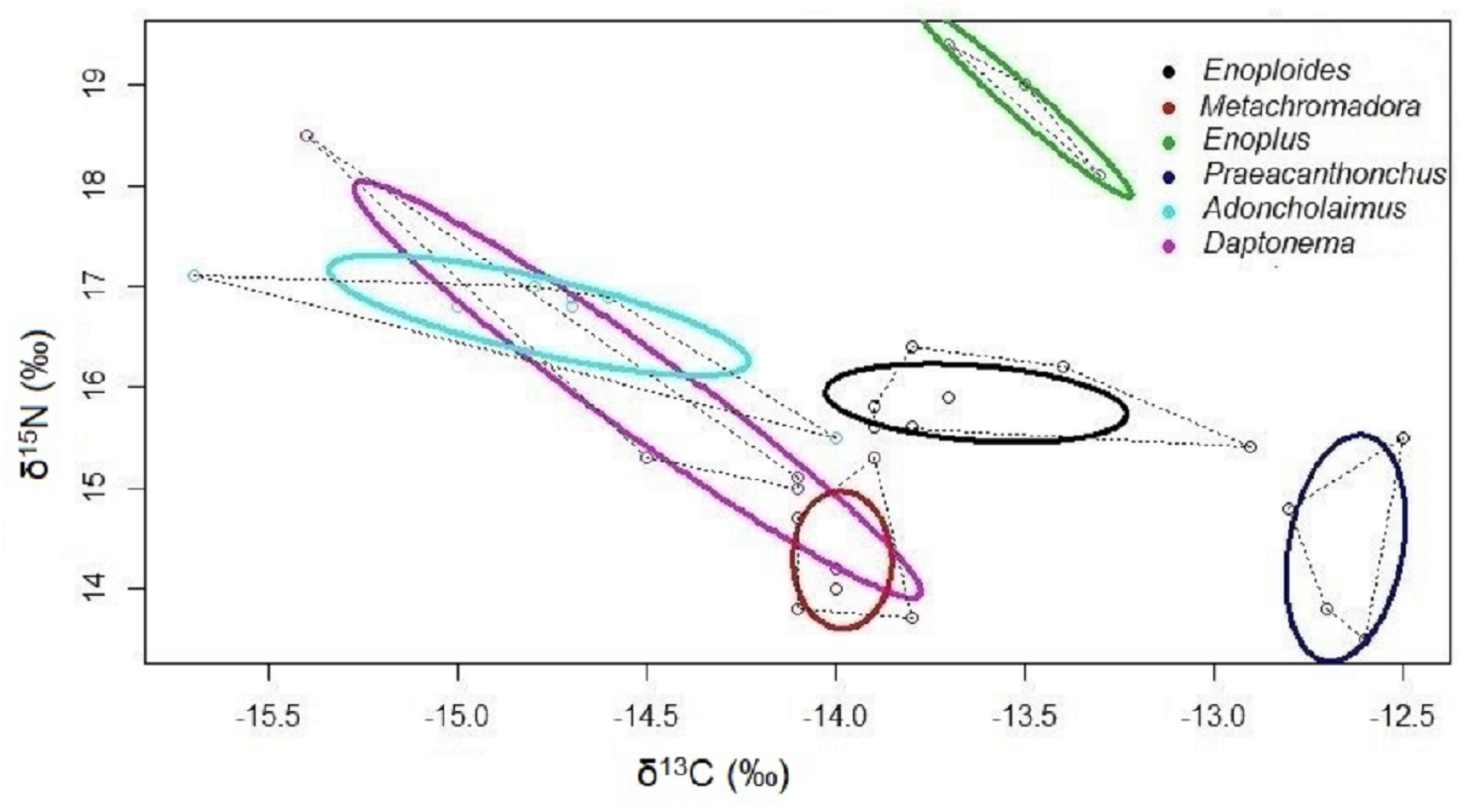
Bivariate isotopic niche spaces of six nematode species from an intertidal flat. Variation in stable isotope composition (*δ*^13^C and *δ*^15^N) of six nematode species, including all replicate samples of each species, irrespective of the exact sampling station in the Paulina. Thick coloured lines are Bayesian bivariate ellipses that represent the core isotopic niche of each nematode species, here based on an inclusion threshold of ∼40% of the data (default value in SIBER). Dotted grey lines are convex hull volumes that depict the smallest bivariate space that includes all data points in the *δ*^13^C/ *δ*^15^N plot. Both the bivariate ellipses and the convex hulls depict the isotopic niches of the six nematode species, and their degree of overlap is inversely proportional to the resource differentiation among the species. Species are indicated by their genus name.

**Figure 4:**
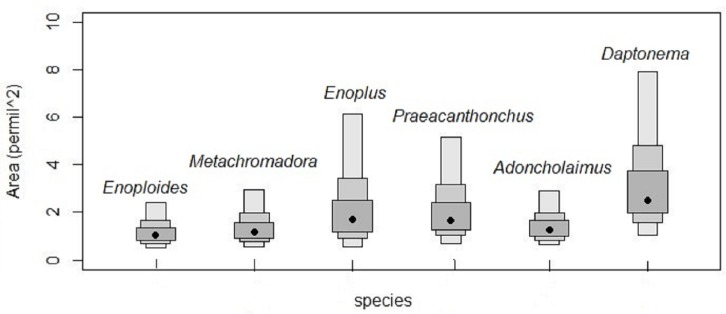
Surface areas of the bivariate isotopic standard ellipses of six nematode species from an intertidal flat. Surface areas of the Bayesian bivariate isotopic standard ellipses depicted in Fig. 3, in units of ‰^2^, because they represent surface areas in an isotopic biplot, where each axis is a *δ* value expressed in ‰. Measures of uncertainty and central tendency (black circles = mode) of standard ellipses are given (SEAc). Boxes show 95, 75 and 50 % credibility intervals from light to dark grey, respectively. Species are indicated by their genus names in the figure.

Isotopic niches based on the stable isotopes of carbon and nitrogen exhibited no overlap between *Enoploides, Enoplus* and *Praeacanthonchus,* nor between any of these three species and the remaining three for which sufficient replicate data were available (Fig. 3). Moreover, *Metachromadora*’s isotopic niche only overlapped with that of *Daptonema* (proportion of overlap = 0.14), and only *Daptonema* and *Adoncholaimus* exhibited a somewhat more pronounced isotopic niche overlap (proportion of overlap = 0.33) (Fig. 3). *Daptonema* also had the largest standard ellipse area, followed by *Enoplus* and *Praeacanthonchus* (Fig. 4), but only the difference in isotopic niche breadth between *Daptonema* and *Enoploides* was statistically significant (with probability = 0.96).

### Fatty acid composition

The fatty acid content and composition of nematodes can be found in supplementary Table S1. In short, total fatty acid (TFA) content ranged from 40 ± 5 ng/ind in *Theristus* to 1,403 ± 213 ng/ind in *Enoplus*, generally exhibiting a clear correlation with individual nematode biomass (Table S1). TFA standardized per unit nematode body mass differed by a factor of 3, with the lowest value in *Enoplus* and the highest in *Oncholaimus* (Table S1). Generally, most nematode species had substantial amounts of PUFA (38% to 64%), with HUFA (34% to 64%) and ω3 PUFA (36% to 59%) being dominant, whereas MUFA (17% to 36%), SFA (12% to 28%) and ω6 PUFA (1% to 6%) were present in lower abundances. Among PUFA, EPA and/or DHA dominated, the sum of these two PUFA ranging from 30% to 54% of total FA. The relative abundance of all these FA classes differed among species (Table S2).

**Figure 5:**
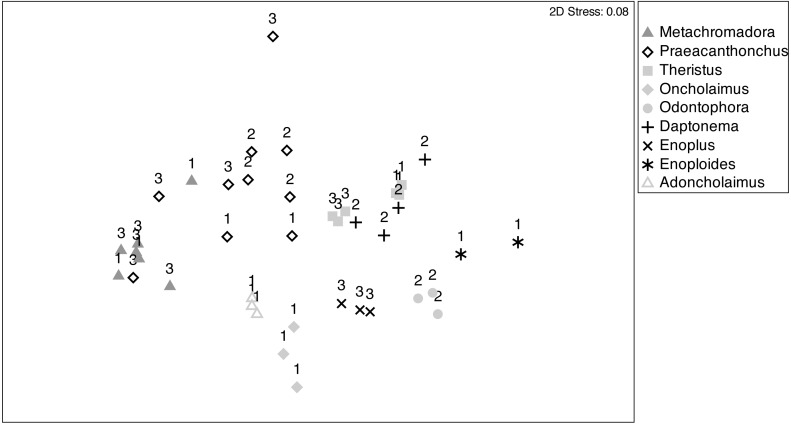
Non-metric multidimensional scaling (nMDS) ordination of nematode fatty acid composition. nMDS ordination of nematode fatty acid composition on the basis of a Bray-Curtis similarity matrix of relative abundances of FAMEs (as % of total fatty acids). Sampling stations are indicated by numbers (1: station 1; 2: station 2; 3: station 3). Numbers of replicates differed between nematode species and sampling stations, depending on the abundance of the species at the time of sampling and on biomass requirements for fatty acid analyses.

Patterns of fatty acid composition among nematode species and stations were visualised in an nMDS ordination (Fig. 5), where the relative distances between samples in the ordination reflect their variation in terms of fatty acid composition. Most pairs of species were differentiated and exhibited fairly limited within-species variability. Species with a presumed partial or main predatory feeding ecology (*Adoncholaimus, Oncholaimus, Enoplus, Enoploides, Odontophora*) had mutually non-overlapping positions in the ordination and were all situated in the lower part of the ordination plot. The two confamiliar xyalid species, *Daptonema* and *Theristus*, had slightly overlapping FA compositions, different from those of all other species, including the other supposed MPB feeders *Metachromadora* and *Praeacanthonchus*. The latter species exhibited by far the largest intraspecific variability, but still had limited overlap with other species (only partly with *Metachromadora*), whereas all except one sample of the former species formed a separate cluster. Of the three species (*Metachromadora, Praeacanthonchus* and *Theristus*) that were obtained from more than one location, only the FA composition of *Theristus* exhibited a slight separation between stations.

The pattern of the nMDS was confirmed by a one-way PERMANOVA with factor nematode species (Table S3), which was highly significant (*df* = 8, Pseudo-F = 16, *p* = 0.001; but note a significant PERMDISP (*p* < 0.05)) and exhibited significant pairwise differences (*p* < 0.05) among all pairs of species. A two-way PERMANOVA with species and station, using only data of the three species which were sampled at two stations (st1, st3), revealed no effect of station nor of station × species, whereas species again had a highly significant effect (*p* = 0.001), with significant differences between all pairs of species. Note, however, that there was a significant dispersion effect, calling for a cautionary interpretation of this species effect.

SIMPER analyses revealed the main FA that contributed to the dissimilarity among species (Table S3). Among the expected MPB feeders, *Metachromadora* was differentiated from *Praeacanthonchus, Daptonema* and *Theristus* mainly by a higher concentration of C16:1ω7 and a lower concentration of DHA. Similarly, *Praeacanthonchus* differed from *Daptonema* and *Theristus* by a lower level of DHA and higher concentrations of EPA, C16:1ω7 and C16:0. *Theristus* had slightly higher concentrations of EPA and C22:1ω9 compared to *Daptonema*, the latter being characterized by a slightly higher concentration of DHA and the presence of C24:1ω9 (Table S3). Presumed MPB feeders differed in many different, species-specific ways from other nematodes, the only nearly consistent difference being the usually higher EPA concentrations and the absence or lower concentrations of C22:5ω3 in MPB feeders. Some other nematodes also had higher concentrations of C18:0 and of ARA (Table S3).

Among these other nematode species, *Oncholaimus* and *Adoncholaimus* both had elevated concentrations of C16:1ω7 and of C16:0 and lower concentrations of DHA compared to most other species (Table S3). *Odontophora* had higher C20:1 and DHA concentrations than other predatory nematodes, except *Enoploides*, which had higher DHA than *Odontophora*. Indeed, *Enoploides* differed from all other presumed predators by its higher levels of DHA. There were no consistent differences between *Enoplus* and other presumed predators.

### Fatty acid composition of tidal flat nematodes

Variation in FA biomarkers among the nine nematode species can be found in Table S2. In short, significant differences were observed in most biomarkers, except the bacterial marker C15:0 + C17:0, and between multiple pairs of nematode species. PERMDISP values were non-significant for most biomarkers, hence the significant differences can be confidently attributed to real factor effects.

Among the diatom biomarkers, EPA concentrations were generally lower in nematodes with presumed predatory feeding than in the presumed MPB feeders *Daptonema, Theristus*, *Metachromadora* and *Praeacanthonchus*; *Praeacanthonchus* had the highest EPA level (30.02 ± 5.38%). Concentrations of C16:1ω7 did not show a similar separation, significantly higher concentrations being found in *Metachromadora* and *Adoncholaimus* compared to all other species. The ratio of C16:1ω7 to C16:0 followed a similar pattern, *Metachromadora* having significantly higher values than all other species, again followed by *Adoncholaimus*. The DHA/EPA ratio was also significantly lower in *Metachromadora* than in all other species. *Praeacanthonchus* and *Adoncholaimus*, in turn, had significantly lower DHA/EPA ratios than the remaining species.

Whereas *Metachromadora* thus consistently scored high values of diatom-related FA biomarkers, it had the significantly lowest concentration of the dinoflagellate marker DHA of all nine species. Highest values for DHA were found in the deposit feeders *Daptonema* and *Theristus* and in the predator/omnivore *Enoploides*. The sum of C14, C16 and C18 was highest in the supposedly predatory/omnivorous *Oncholaimus* and *Adoncholaimus*, followed by *Enoploides* and *Metachromadora*. The lowest values were present in the deposit-feeding species *Daptonema* and *Theristus* and in *Odontophora*.

Most species had negligible concentrations (<2.5%) of C_18_PUFA, indicating a limited—if any—contribution of vascular plant detritus. Only *Metachromadora* had a C_18_PUFA concentration >2%, while this marker was completely absent from *Enoploides*.

The bacterial biomarker C15:0+C17:0 ranged from 2.95 ± 0.63% in *Enoploides* to 9.8 ± 3.24% in *Oncholaimus*, but without a significant species effect. Similarly, no significant differences were observed among species in the proportion of C24:0.

Markers of carnivory did not reach high relative abundances, but did differ significantly between species. C20:1ω9 always comprised <4% of TFA, with highest values in *Enoploides* and *Enoplus* and lowest in *Oncholaimus*. The former two species and *Odontophora* generally had significantly higher levels of this FA than all other species (Table S2). The ratio of PUFA/SFA was lowest in *Metachromadora* but highest in the two Xyalidae, rather than in any presumed predatory species. Still, differences between the Xyalidae, *Enoploides, Enoplus* and *Odontophora* were not statistically significant (Table S2).

When focusing on the two-way comparison of stations (2 levels) and species (3 levels) (Table S5), no significant differences were observed in the relative abundance of EPA among stations, species or their interaction (see Table S5). Another diatom marker, C16:1ω7, only differed among species, while the ratio of C16:1ω7/C16:0 was significantly affected by the interaction of species x station: *Metachromadora* and *Praeacanthonchus* had higher values of this ratio at st3 than at st1, while *Theristus* showed the opposite pattern. The ratio DHA/EPA was lowest in *Metachromadora*, followed by *Praeacanthonchus* and *Theristus*. It was also significantly lower in st3 than in st1 (Tables S1 and S5).

The relative concentration of DHA and of the bacterial marker C15:0 + C17:0 did not differ between stations nor species (Tables S1 and S5).

## Discussion

### Carbon sources of tidal flat nematodes

As in previous studies on estuarine tidal flats and coastal beaches (Carman & Fry, 2002; Moens, Bouillon & Gallucci, 2005; Moens et al., 2002; Moens et al., 2014; Rzeznik-Orignac et al., 2008; Maria et al., 2011), microphytobenthos was the predominant basal carbon source for the majority of nematode species in this study, as evidenced by the relatively ‘heavy’ carbon isotopic signatures of all species except *Oncholaimus* (Moens et al., 2002). The *δ*^13^C of *Oncholaimus* was relatively depleted compared to our MPB measurements, suggesting some contribution of other resources. These may include settled suspended particulate matter (Boschker, Kromkamp & Middelburg, 2005; Hellings et al., 1999), although sources like macroalgae cannot be excluded (Riera & Hubas, 2003).

Interestingly, *Oncholaimus* had the largest proportion of arachidonic acid, an indicator of microzooplankton (Parrish et al., 1995). Given its ability to prey on small invertebrates (Moens & Vincx, 1997) and to scavenge on dead animals (Jensen, 1987), it is possible that dead zooplankton contributed to its diet. Substantial quantities of marine zooplankton enter the Schelde Estuary at high tide and die there, yielding ca 1,500 tonnes dry weight of dead marine zooplankton which decays in the estuary per year (Soetaert, Herman & Kromkamp, 1994); much of this dead zooplankton ends up in sediments and could contribute to the nutrition of benthic animals. On the other hand, this was not clearly reflected in the concentrations of the FA’s C20:1 and C20:2, indicators of feeding on (macro)zooplankton in estuaries (Parrish et al., 2000), in *Oncholaimus* (3.96 ± 0.41%). Since the *δ*^13^C of *Oncholaimus* is based on a single sample, albeit composed of tens of individuals, we cannot draw firm conclusions. It is nevertheless noteworthy that the pigment pyropheophytin, which is commonly used as an indicator of zooplankton faecal pellets (Wright & Jeffrey, 1997), was an important driver of total nematode abundance and nematode genus composition at the Paulina intertidal flat (Wu et al., 2019), suggesting that the potential of zooplankton-related inputs as a resource to estuarine nematodes deserves further investigation.

MPB biofilms on tidal flats in the Schelde Estuary are commonly dominated by diatoms (Sabbe & Vyverman, 1991; Hamels et al., 1998). Our fatty-acid data nevertheless suggest variable contributions of other microalgae, particularly dinoflagellates, to the diets of nematodes, in line with results obtained for two harpacticoid copepod species (Cnudde et al., 2015). The significance of dinoflagellates is evidenced by DHA concentrations that rivalled those of EPA in five out of the nine nematode species studied here.

In this context, it is tempting to explain the relatively heavy *δ*^13^C of *Praeacanthonchus* as an indication that it may utilize different components of the MPB than other nematodes do, such as dinoflagellates, which at station 1 regularly form a significant component of biofilms (T Moens, pers. obs., 1998–2014). However, there was no obvious match in our data between nematode *δ*^13^C and the proportion of dinoflagellate marker FA, nor did the concentration of peridinin (a light-harvesting pigment characteristic of dinoflagellates) explain a significant portion of the spatial variability in nematode assemblages at the Paulina tidal flat (Wu et al., 2019).

Moens et al. (2014) suggested that the large trophic fractionation between MPB and presumed MPB grazers could represent a real value, despite fractionation factors (FF) usually being lower at lower TL’s (McCutchan et al., 2003). Alternatively, this large FF may indicate that part of the MPB carbon is obtained through a trophic intermediate. Our results suggest that bacteria are unlikely to be that intermediate, mainly because bacterial marker FA’s were present in limited abundances in all nematode species (see below). Certain heterotrophic protists might provide an alternative explanation (Leduc, 2009), but in the absence of good protozoan biomarkers, we can only speculate on this.

Our FA data provide evidence against the idea that bacteria would contribute a major share (Pascal et al., 2009) to the diet of tidal-flat nematode species, since the bacterial markers C15:0 and C17:0 together always comprised <4% of nematode TFA. Adding C18:1ω7 raised the proportion of bacterial FA to between 5.1 (*Enoploides*) and 11.8% (*Oncholaimus*), compared to, for instance, 38 to 57% for the sum of the microalgal markers EPA, DHA and C16:1ω7. Part of these bacterial FA may actually reflect various kinds of nematode-bacteria cohabitations (such as gut bacteria) which collectively form a nematode’s microbiome (Derycke et al., 2016). This is supported by the fact that the bacterial marker FA contributions did not differ substantially between species with different feeding modes. Whereas we expected higher bacterial contributions in nematodes which ingest particles whole rather than piercing them and sucking out the contents, we found higher contributions of bacterial markers in the epistrate feeding *Metachromadora* than in deposit feeders or the omnivore *Enoploides*. Low contributions of bacterial FA’s were also found in a majority of the harpacticoid copepod species on the same tidal flat (Cnudde et al., 2015). Discrepancies between isotope and FA data (Cnudde et al., 2015) nevertheless indicate that caution is due when drawing conclusions about the (lack of) importance of bacteria in the diet of tidal-flat meiofauna.

Our SI- and FA-based interpretations on the diet and trophic position of nematodes can be challenged because of methodological limitations inherent to the use of SI and FA in the reconstruction of food webs. The main drawback to the use of FA is the ability of consumers like nematodes to produce their own FA. Hence, not all FA derive directly from their resources (Schlechtriem et al., 2004; Leduc & Probert, 2009), and nematode species sharing the same resource may still exhibit differences in their FA composition (Hutzell & Krusberg, 1982). Variation in environmental factors may further result in variation in consumer FA despite food availability remaining constant. In addition, not all marker FA used here are equally well established proxies of particular organisms/sources (Peterson et al., 2005). On the other hand, there is considerable uncertainty about the variation in trophic level fractionation for N isotopes within and between consumer species. The traditional idea that *δ*^15^N differs by 3.4‰ between a consumer and its resource (Post, 2002) cannot be generalized; the TFF may range from <2 to >5 (McCutchan et al., 2003), and it remains to be established whether the TFF relates to trophic level.

### The nematode part of the benthic food web comprises more than two trophic levels and a substantial degree of omnivory

The nitrogen isotopic ratios of MPB and nematodes immediately reveal that MPB is not always directly consumed by all nematode species. This is not a novel result, yet the trophic structure of this study’s small ‘food web’ reveals some striking features.

**Figure 6:**
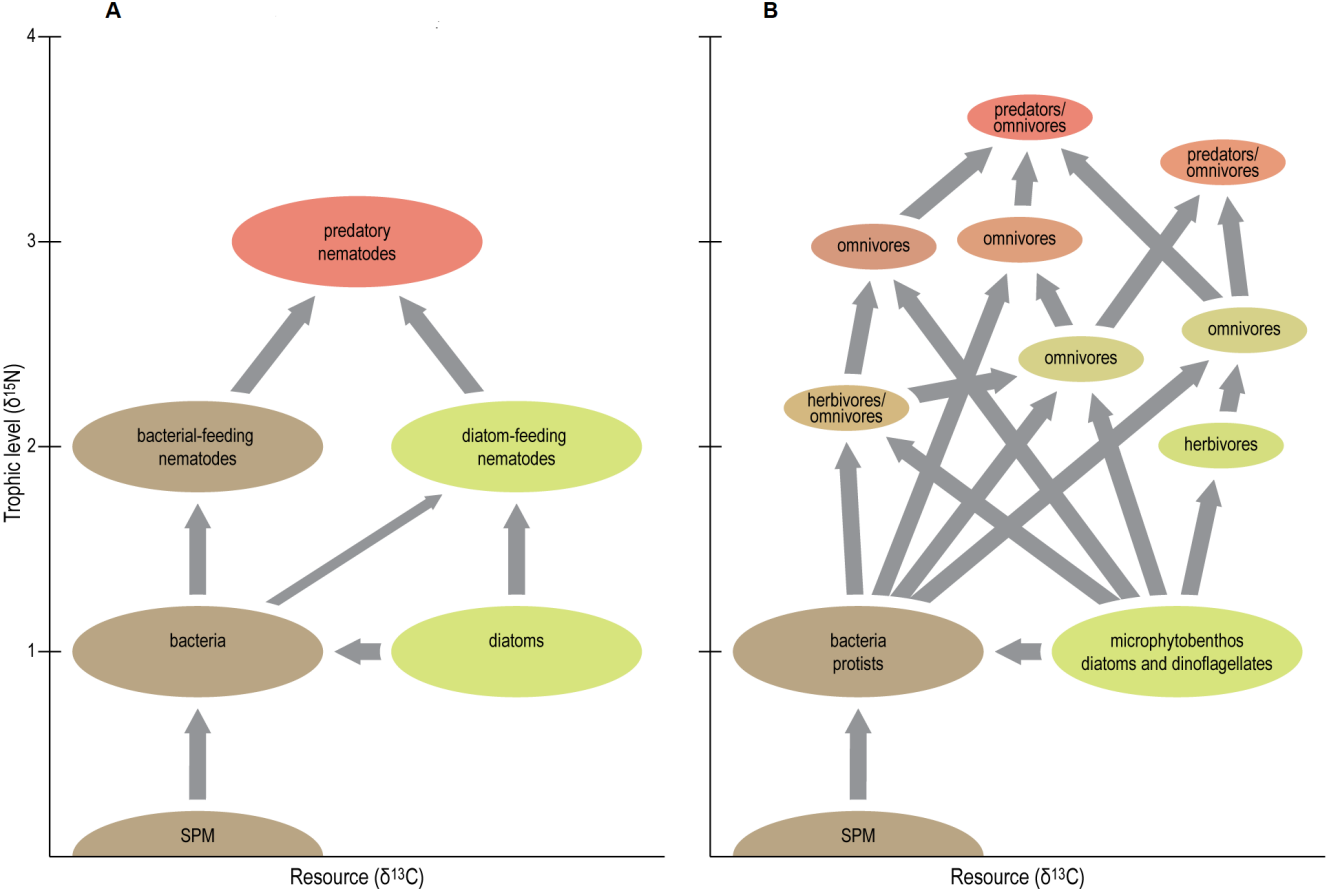
Schematic view of traditional (A) vs present (B) position of nematodes in tidal-flat food web. Figure conceived by Tim ‘tKint. Conceptual scheme of the positions of nematodes in tidal-flat foodwebs. A: simplified ‘old’ view where most nematodes are either grazers of microalgae or bacteria, or predators of these grazers, and hence occupy distinct trophic levels (i.e., primary and secondary consumers, respectively). B: new view based on the results of the present study. Apart from some grazers of microalgae (i.e., herbivores), most nematodes exhibit omnivory and thus occupy intermediate trophic levels. The food web is more complex, and some predatory/omnivorous nematodes exhibit a trophic level above that of secondary consumers. Resource differentiation among species is pronounced, and resource niche spaces are of similar size, irrespective of the trophic level of the species. Note that arrows represent presence/absence of particular trophic links but do not indicate the magnitude of the corresponding carbon flows.

First of all, the idea that most nematodes are either primary consumers, grazing on MPB, or predators foraging on primary consumers, is too simple (Fig. 6). Trophic-level calculations rather suggest that the nematodes studied here span up to three trophic levels. Under the assumption that *Metachromadora* is a primary consumer (i.e., TL = 2), *Enoplus* has a TL of 3.3 or 3.8 in case of a fractionation factor of 3.4 or 2.5‰, respectively. *Odontophora* follows with respective TL’s of 3.2 and 3.6. Gut content observations on *Enoplus* revealed that it is a generalist feeder, ingesting prey ranging from cyanobacteria and diatoms up to rotifers and oligochaetes (Hellwig-Armonies, Armonies & Lorenzen, 1991). The elevated *δ*^15^N of the species in this and a previous study (Moens, Bouillon & Gallucci, 2005) suggest that it obtains a dominant share of its diet from preying on a combination of species belonging to the second and third trophic level, or on omnivorous prey species.

In any case, our results underline the presence of multiple trophic levels in estuarine nematode assemblages (Fig. 6), thus largely invalidating whole-community estimates of trophic level, which have been relatively common because of the difficulty in obtaining sufficient nematode biomass for species- or genus-level analyses. They also convincingly demonstrate that *Odontophora* is not a deposit feeder, but ranks among the highest TL’s in estuarine nematode communities.

A second conclusion from our *δ*^15^N results is that omnivory is common in estuarine nematodes. With few exceptions, estimated TL’s of nematodes had non-integer values, indicating that they obtain resources from more than one trophic level (Fig. 6). Notable exceptions were *Metachromadora, Praeacanthonchus* and *Theristus* in three out of the four scenarios, *Adoncholaimus* when applying a TFFof 2.5‰, and *Enoploides* and *Oncholaimus* in one scenario each.

The trophic position of *Praeacanthonchus*, level with that of *Metachromadora* and *Theristus*, differs from its omnivorous position in between the latter two species and species at higher trophic levels in a previous study at the Paulina tidal flat (Moens et al., 2014). This suggests that *Praeacanthonchus* may be an opportunistic feeder which can temporarily switch resources depending on their availability and/or on competitive interactions. The giant deep-sea nematode *Deontostoma tridentum* exhibited an even much more pronounced variability in trophic level, which spanned 1–3 units (Leduc et al., 2015). However, because of its much larger size, the results on *Deontostoma* were obtained on single individuals and thus represent interindividual variation, whereas each of our *Praeacanthonchus* samples was composed of many tens of specimens.

Depending on the precise scenario, *Oncholaimus, Adoncholaimus, Enoploides* and *Daptonema*, in order of decreasing TL, together spanned almost one trophic level above the three abovementioned primary consumers, pointing at omnivorous feeding strategies with different relative contributions of predation vs primary consumption. *Daptonema* is closely related to *Theristus*, but at least this species (*D. hirsutum*) has a larger body and mouth size, allowing it to access resources that are unavailable to *Theristus*. While *Daptonema* has often been observed with diatom frustules in its intestine (Moens & Vincx, 1997; Nehring, 1992), it can also swallow small nematodes as part of a food selection strategy that is mainly based on particle size and shape (Moens & Vincx, 1997). *Enoploides* is a voracious predator of prey ranging from ciliates to nematodes and oligochaetes (Gallucci, Steyaert & Moens, 2005; Hamels et al., 2001; Moens & Vincx, 1997; Moens et al., 2000), yet it also ingests microalgae (Franco et al., 2008; Moens et al., 2014). The present TL results indicate that *Enoploides* obtained roughly equal amounts of carbon from the first and second trophic level. Both oncholaimid species were previously classified as facultative predators (Moens & Vincx, 1997) or scavengers (Jensen, 1987). In the case of *Adoncholaimus*, its high scores of the diatom markers C16:1ω7 and C16:1ω7/C16:0 and its intermediate contribution of EPA suggest that it too may obtain part of its food by grazing on diatoms and/or by preying on diatom grazers. Based on their FA profiles, *Adoncholaimus* and *Oncholaimus* were the secondmost similar pair of species, with a similarity of 85%. The two species differed mainly in their concentrations of the diatom markers C16:1ω7 and EPA (higher in *Adoncholaimus*), of arachidonic acid and of C22:1ω9 (both indicative of feeding on zooplankton and higher in *Oncholaimus*). These slightly more ‘diatom-oriented’ and ‘carnivory-oriented’ FA profiles in *Adoncholaimus* and *Oncholaimus*, respectively, are in accordance with the slightly higher TL of the latter species.

Thirdly, the isotopic niche size of nematodes did not clearly correlate with trophic level nor did it match well with presence of omnivory. The only significant difference in bivariate standard ellipse areas occurred between *Daptonema* (largest SEA) and *Enoploides* (smallest SEA), two species which in the present study exhibited substantial omnivory and had fairly similar TL’s. Hence, our data indicate that most nematode species utilized different resources, and that the degree of resource variability did not strongly differ between species.

Finally, neither the ratio of PUFA/SFA nor the abundance of 20:1ω9 appeared reliable indicators of carnivory, since they did not correlate with trophic level. PUFA/SFA values were highest in the two species of Xyalidae, which both had relatively low TL. 20:1ω9 was highest in carnivorous/omnivorous species, mainly *Enoplus* and *Enoploides*, suggesting that it may be a useful marker in some cases, but it had its lowest values in the two species of Oncholaimidae, which exceeded *Enoploides* in trophic level.

### Resource differentiation among nematode species is prominent

In intertidal flats, the diversity and small-scale patchiness of resources, as well as the temporal variation in their availability, combined with species-specific feeding preferences, offer a basis for resource-driven niche differentiation (Azovsky et al., 2005; Pace & Carman, 1996).

We determined bivariate core isotopic niche areas for six nematode species, which were *a priori* assigned as predators/omnivores (three species: *Enoplus, Enoploides, Adoncholaimus*), deposit feeders (two species: *Daptonema, Praeacanthonchus*) and epistratum feeders (*Metachromadora*). It is important to stress that deposit feeders and epistratum feeders can both feed on microalgae (Moens, Yeates & De Ley, 2004). The core isotopic niches of the three herbivore species differed strongly: that of *Praeacanthonchus* was completely separate from both other species, whereas there was limited overlap between those of *Daptonema* and *Metachromadora*. Even though core isotopic niche spaces do not depict the entire resource niche space, this result demonstrates that these three species differed significantly in their resource use. Different size fractions of diatom biofilms can exhibit different isotopic signatures (Rzeznik-Orignac et al., 2008), and food-particle size has repeatedly been demonstrated to be an important driver of feeding selectivity in meiofauna (De Troch et al., 2006; Moens et al., 2014). *Daptonema* and *Praeacanthonchus* had very different DHA/EPA and C16:1ω7/C16:0 ratios, both indicating that *Praeacanthonchus* fed more on diatoms, whereas dinoflagellates contributed substantially to the diet of *Daptonema*. The slightly higher TL and core ellipse area of *Daptonema* suggest that this species has additional feeding strategies which *Praeacanthonchus* and *Metachromadora* lack. The FA profile of *Metachromadora* strongly supports a preference for diatoms, which seems at odds with results on *Metachromadora* from an intertidal site with *Zostera marina* vegetation, where it probably fed on *Zostera* detritus-associated bacteria and/or fungi (Vafeiadou et al., 2014). It is possible that this species also scrapes off bacteria from microalgal cells or sediment grains, which would be consistent with *Metachromadora* having the secondhighest proportion of bacterial marker FA of all species in this study.

Like the herbivores, supposedly carnivorous nematodes had non-overlapping core standard ellipse areas. *Enoplus* was separated from *Enoploides* and *Adoncholaimus* by its higher trophic level, whereas the latter two species were mainly differentiated by different carbon isotope signatures, suggesting they utilize partly different resources. The high proportion of DHA and low levels of C16:1ω7 in *Enoploides* indicate that this species used dinoflagellates. *Enoploides* is clearly an opportunistic feeder (see above). The isotopic niche space of *Adoncholaimus* substantially overlapped with that of *Daptonema* rather than with other carnivorous species, but exhibited a higher mean TL. Based on FA profiles, these two species were mainly differentiated by a stronger diatom signal in *Adoncholaimus* vs a more pronounced dinoflagellate imprint in *Daptonema*.

Pairwise dissimilarities in nematode fatty acid composition ranged from 14–15% between the two species of Xyalidae (*Daptonema* and *Theristus*) and between the two Oncholaimidae (*Oncholaimus* and *Adoncholaimus*) to 49% between *Enoploides* and *Metachromadora*. Hence, the two species pairs with the most similar FA composition both comprised two confamiliar species. This could be because more closely related species have a more similar feeding ecology, but also because they have a more similar physiology/metabolism. Given that the isotopic niches in both species pairs were different, the latter explanation may be important.

nMDS separated the supposedly carnivorous species in the lower half of the plot from the other species. Most striking, however, was the exceptionally low overlap between species, with the two Xyalidae on the one hand, and *Metachromadora* and *Praeacanthonchus* on the other, forming the only two species pairs which exhibited some mutual overlap.

Our data thus support the importance of resource differentiation among both distantly and closely related nematode species. Such niche differentiation may seem at odds with the considerable flexibility in feeding behaviour in nematodes (Moens, Yeates & De Ley, 2004). However, in a spatially and temporally highly dynamic environment, niche properties cannot be viewed as static (Vandermeer, 1972; Amarasekare, 2003), and they also depend on the competitive environment in which a population operates. Depending on the competitive regime, individuals and populations may exhibit temporary niche shifts (here: shifts in resource use) or niche contractions (here: a narrower resource use) to reduce competition within and between populations (MacArthur & Levins, 1964; Svanbäck & Bolnick, 2007). A flexible resource strategy is therefore not at odds with resource differentiation (Ashton et al., 2010). Our results further highlight the limits of traditional black-box approaches, in which most meiofaunal species are considered primary consumers, and of feeding-guild classifications, which create at least partly artificial groupings of species which in reality have a substantially different feeding ecology and trophic level.

## Conclusion

Using a combination of natural stable-isotope ratios of carbon and nitrogen and of fatty-acid composition of nine nematode species from an estuarine intertidal flat, we provide evidence that resource differentiation is pronounced among as well as within feeding modes and resource guilds. Nematodes comprise up to three different trophic levels (from primary to tertiary consumers), yet with the exception of some herbivores, omnivory is prominent (Fig. 6). Bivariate isotopic niche spaces were of similar size among most species, irrespective of their trophic level (Fig. 6). Herbivory importantly contributes to the nutrition of herbivores as well as carnivores; it mainly targets diatoms in some species, yet prominently includes dinoflagellates in others. Bacteria, in contrast, appear to be of limited nutritional importance. *Odontophora setosus* is identified as a predator/omnivore with a trophic level in between that of secondary and tertiary consumers.

##  Supplemental Information

10.7717/peerj.7864/supp-1 Data S1 Raw data

10.7717/peerj.7864/supp-2 Supplemental Information 2 Supplemental Tables

## Additional Information and Declarations

### Data Availability

The following information was supplied regarding data availability: The raw measurements are available in the Supplemental File.
