# Supplementary material for: Natural stable isotope ratios and fatty acid profiles of estuarine tidal flat nematodes reveal very limited niche overlap among co-occurring species
Source: PeerJ. 2019 Oct 11;7:e7864. doi: 10.7717/peerj.7864 (PMC6791341; doi:10.7717/peerj.7864)
Supplement: Supplemental Information 2 [file peerj-07-7864-s002.docx]

# Supplementary information

Table S1. **List of fatty acid concentrations.**

Concentrations of total fatty acids (TFA: ng/nematode; TFA/biomass: ng/µm^3^), relative concentrations of specific fatty acids or FA classes (%), and values of some FA ratios with biomarker value in nine estuarine tidal flat nematode species. *M, P, T, D, On, Od, Enoplu, Enoplo* and *A* indicate the nematode species: *Metachromadora remanei, Praeacanthonchus punctatus, Theristus acer, Daptonema hirsutus, Oncholaimus oxyuris, Odontophora setosus, Enoplus brevis, Enoploides longispiculosus,* and *Adoncholaimus fuscus*, respectively.

| Variables | *M* | *P* | *T* | *D* | *On* | *Od* | *Enoplu* | *Enoplo* | *A* |
| --- | --- | --- | --- | --- | --- | --- | --- | --- | --- |
| TFA | 61±19 | 85±56 | 40±5 | 70±22 | 255±30 | 53±6 | 1403±213 | 362±100 | 1089±89 |
| TFA/ biomass | 206.8 | 131.7 | 174.6 | 123.9 | 262.6 | 226.1 | 97.3 | 129.5 | 121.9 |
| PUFA | 39.5±4.7 | 51.5±6.9 | 60.8±3.5 | 60.8±4 | 44.8±3.9 | 56.5±2.7 | 55.6±1.2 | 59.7±2.3 | 44.1±0.7 |
| HUFA | 34.6±4.7 | 48.1±8.5 | 60.5±3.4 | 60.7±4 | 43.9±3.9 | 55.6±2.7 | 53.4±1.1 | 58.1±2.2 | 41.5±0.6 |
| MUFA | 34.9±5.4 | 23±6.5 | 23.6±1 | 24.1±3.4 | 27±4.1 | 24.5±1 | 23.1±0.1 | 17.2±1.5 | 29.2±0.4 |
| SFA | 25.7±1.3 | 25.5±4.7 | 15.7±3.7 | 15.1±3 | 28.2±2.4 | 19.1±2.8 | 21.3±1.2 | 23.2±0.7 | 26.8±1.1 |
| ω3pufa | 37±4.7 | 49.1±7.6 | 56.9±2.9 | 57.4±3.6 | 36.5±2.9 | 49.9±2.3 | 47.6±0.9 | 55±3.4 | 37±0.4 |
| ω6pufa | 2.5±0.6 | 2.3±1.8 | 3.8±0.7 | 3.5±0.7 | 7.9±0.9 | 6.6±0.4 | 6.5±0.2 | 3.1±1.2 | 6.3±0.4 |
| C18PUFA | 2.2±0.7 | 1.3±0.8 | 0.3±0.2 | 0.1±0.3 | 0.5±0 | 0.9±0.1 | 0.7±0.1 | - | 1.2±0.1 |
| EPA | 25.5±2.6 | 30±5.4 | 26.8±0.6 | 24.2±2.3 | 18.3±1.5 | 17.7±0.8 | 19.6±0.6 | 17±1.4 | 21.2±0.7 |
| DHA | 7.4±1.7 | 14.2±3.7 | 24.6±2.7 | 28.5±5.5 | 11.7±0.8 | 21.1±0.9 | 16.1±0.9 | 28±3.4 | 10.1±0.5 |
| EPA/DHA | 3.5±0.4 | 2.3±0.6 | 1.1±0.1 | 0.9±0.3 | 1.6±0.1 | 0.8±0 | 1.2±0.1 | 0.6±0.1 | 2.1±0.1 |
| C16:1ω7 | 18.5±4.2 | 8.4±5.3 | 4.8±0.6 | 4±1.4 | 8.2±1.3 | 4±0.5 | 5.4±0.3 | 2.3±1.2 | 12.8±0.4 |
| C16:1ω7/c16:0 | 1.5±0.3 | 0.8±0.4 | 0.6±0.1 | 0.6±0.2 | 0.6±0.1 | 0.7±0 | 0.6±0.1 | 0.3±0.1 | 0.9±0 |
| C14:0+C16:0+C18:0 | 20.1±1.2 | 19.3±4 | 14.5±3.9 | 13.3±2.9 | 26.1±2.4 | 13.3±0.8 | 18.3±1.2 | 20.8±0.6 | 24.2±1.2 |
| C15:0+C17:0 | 3.2±1.4 | 2.5±2 | 0.9±0.1 | 0.9±0.1 | 2±0.2 | 1.2±0 | 2.6±0.2 | 2.2±0.1 | 2±0.1 |
| C18:1ω7 | 8.1±0.9 | 6.5±1.2 | 6.1±1 | 7.7±0.4 | 9.8±3.2 | 6±0.5 | 7.9±0.1 | 3±0.6 | 7.7±0.3 |
| C20:1+C22:1 | 2.5±0.8 | 2.3±0.9 | 6.5±1 | 4±0.1 | 4±0.4 | 5±0.4 | 2.5±0.1 | 5.7±0.2 | 2.9±0.1 |
| ARA | 0.4±1 | 0.6±1.1 | 3.3±0.4 | 3.2±0.1 | 7.5±0.9 | 5.7±0.5 | 5.3±0.3 | 2.8±0.8 | 4.7±0.3 |
| C24:0 | 0.1±0.1 | 0.1±0.1 | - | - | - | 0.1±0 | - | - | - |
| C18:1ω9 | 0.2±0.6 | 0.5±0.8 | 3.4±0.2 | 2.7±0.4 | 4±2.7 | 4.1±0.1 | 2.3±0.3 | 0.5±0.1 | 3.2±0.2 |
| LC-SCF(C20-24) | 2.4±0.7 | 3.6±0.6 | 0.3±0.2 | 1±0.3 | - | 4.6±2.9 | 0.5±0 | 0.3±0 | 0.6±0 |
| c18:2ω6 | 1.3±0.3 | 0.9±0.6 | 0.3±0.2 | 0.1±0.3 | 0.5±0 | 0.9±0.1 | 0.7±0.1 | - | 0.9±0.1 |
| 20:3ω3 | - | 0.1±0.2 | 0.6±0.1 | 0.9±0 | 1.5±0.2 | 1.8±0.2 | 2.4±0.2 | 1.6±0.1 | 0.7±0.9 |
| 20:3ω6 | 0.1±0.2 | 0.1±0.2 | 0.2±0.2 | 0.2±0.3 | - | - | 0.6±0.1 | 0.3±0.5 | 0.2±0 |
| C14:0 | 1.6±0.2 | 1.5±0.6 | 1±0.2 | 0.8±0.2 | 1.5±0.1 | 1.1±0.1 | 1.2±0.2 | 0.7±0.2 | 1.6±0.2 |
| C15:0 | 2.4±1.3 | 1.5±1.8 | - | - | 0.8±0.1 | 0.3±0 | 1.5±0.1 | 1.1±0 | 1.3±0.1 |
| C15:1ω5 | 0.7±0.9 | 0.8±1.5 | 0.7±0.2 | 0.6±0.1 | 1±0.1 | 0.5±0.1 | - | - | - |
| C16:0 | 12.7±1 | 11±2.7 | 9±2.5 | 7.1±1.5 | 14.2±0.5 | 5.5±0.5 | 9.1±1 | 6.7±0.7 | 13.6±0.1 |
| C16:2ω6 | 0.7±0.4 | 0.7±0.5 | - | - | - | - | - | - | 0.6±0.1 |
| C16:3ω3 | 1.9±0.9 | 1.4±0.9 | - | - | - | - | - | - | 0.4±0.1 |
| C17:0 | 0.8±0.2 | 1±0.3 | 0.9±0.1 | 0.9±0.1 | 1.2±0.1 | 0.9±0 | 1±0 | 1.1±0.1 | 0.7±0 |
| C17:1ω7 | 2.7±1.5 | 2.4±4.6 | 0.2±0.2 | 0.3±0.4 | - | 0.3±0.1 | 0.5±0 | 0.1±0 | 0.3±0 |
| C18:0 | 5.8±0.5 | 6.8±1.3 | 4.6±1.3 | 5.5±1.3 | 10.4±2 | 6.6±0.4 | 8±1.2 | 13.3±0.2 | 9±1.2 |
| C18:2ω6cis | 0.6±0.3 | 0.4±0.4 | - | 0.1±0.3 | - | 0.5±0 | - | - | 0.3±0 |
| C18:2ω6tr | 0.8±0.4 | 0.5±0.5 | 0.3±0.2 | - | 0.5±0 | 0.4±0.1 | 0.7±0.1 | - | 0.6±0.1 |
| C18:3 | 0.1±0.1 | 0.1±0.1 | - | - | - | - | - | - | 0.3±0 |
| C18:4ω3 | 0.8±0.4 | 0.3±0.5 | - | - | - | - | - | - | - |
| C20:0 | 1±0.2 | 1.7±0.4 | 0.3±0.2 | 0.6±0.1 | - | 3.3±2.9 | 0.3±0 | 0.3±0 | 0.2±0 |
| C20:1 | 0.6±0.1 | 0.3±0.3 | 1.3±0.3 | 1.7±0.1 | 0.9±0.1 | 4.7±0.1 | 1.6±0.1 | 1±0 | 1.7±0.1 |
| C20:2 | - | - | - | - | 0.4±0.4 | - | 1.4±0.1 | 1.6±0.1 | 0.5±0.1 |
| C21:0 | 0.7±0.1 | 0.2±0.3 | - | - | - | - | - | - | - |
| C22:0 | 0.7±0.4 | 1.7±0.5 | - | 0.4±0.2 | - | 1.2±0 | 0.2±0 | - | 0.4±0 |
| C22:1ω9 | 2±0.7 | 2.1±1 | 5.2±0.7 | 2.3±0.2 | 3.1±0.4 | 0.3±0.3 | 0.9±0 | 4.7±0.2 | 1.2±0 |
| C22:5ω3 | 1.3±0.5 | 3.1±1 | 4.9±0.4 | 3.7±1.2 | 5±0.5 | 9.2±0.5 | 9.5±0.7 | 8.4±1.5 | 4.6±0.2 |
| C24:1ω9 | 0.1±0.3 | 0.3±0.4 | 0.1±0.3 | 2.7±2.8 | - | 1.6±0.1 | 0.9±0.1 | 2.3±0.1 | 0.4±0.4 |
| C16:2+C16:3 | 2.7±1.2 | 2.1±1.2 | - | - | - | - | - | - | 1±0.1 |
| C16:1ω7+C18:1ω7 | 26.6±4.9 | 14.9±6.3 | 10.9±1.5 | 11.6±1.7 | 18±2 | 10±1 | 13.3±0.3 | 5.2±1.8 | 20.5±0.2 |
| C20:1ω9 | 2±0.1 | 1.9±0.7 | 1.9±0.2 | 2.2±0.1 | 0.1±0.2 | 2.9±0.1 | 3.6±0.3 | 3.4±0.1 | 1.8±0.2 |
| DHA/EPA | 3.5±0.4 | 2.3±0.6 | 1.1±0.1 | 1.2±0.3 | 0.6±0 | 1.2±0 | 0.8±0.1 | 1.7±0.3 | 0.5±0 |
| PUFA/SFA | 1.5±0.2 | 2.1±0.7 | 4.1±1.2 | 4.2±1 | 1.6±0.2 | 3±0.6 | 2.6±0.2 | 2.6±0.2 | 1.7±0.1 |

values lower than 1 % are marked with -.

Table S2. **Results of PERMANOVA on dataset of different FA classes and biomarkers in nine species of nematodes.**

Species are indicated by their genus name; *A* presents the results of the main tests and of PERMDISP tests, while *B* shows the results of pairwise tests.

A

| Variables | df | SS | MS | Pseudo-F | P(perm) | p_PERMDISP |
| --- | --- | --- | --- | --- | --- | --- |
| TFA | 8 | 7150500 | 893820 | 190.2 | ***0.001*** | 0.022 |
| PUFA | 8 | 0.24 | 0.03 | 13.404 | ***0.001*** | 0.356 |
| HUFA | 8 | 0.34 | 0.04 | 14.457 | ***0.001*** | 0.163 |
| MUFA | 8 | 0.09 | 0.01 | 5.7036 | ***0.002*** | 0.23 |
| SFA | 8 | 0.08 | 0.01 | 9.9993 | ***0.001*** | 0.701 |
| ω3PUFA | 8 | 0.26 | 0.03 | 13.087 | ***0.001*** | 0.057 |
| ω6PUFA | 8 | 0.015 | 0.0019 | 15.674 | ***0.001*** | 0.377 |
| C18PUFA | 8 | 0.002 | 0.0003 | 9.5388 | ***0.001*** | 0.481 |
| EPA | 8 | 0.08 | 0.01 | 9.9 | ***0.001*** | ***0.008*** |
| DHA | 8 | 0.21 | 0.03 | 29.7 | ***0.001*** | 0.131 |
| EPA/DHA | 8 | 35.6 | 4.5 | 28.7 | ***0.001*** | 0.397 |
| C16:1ω7 | 8 | 0.11 | 0.01 | 11.63 | ***0.001*** | 0.533 |
| C16:1ω7/C16:0 | 8 | 4.3 | 0.5 | 10 | ***0.001*** | 0.657 |
| C14:0+C16:0+C18:0 | 8 | 0.06 | 0.007 | 8.8 | ***0.001*** | 0.473 |
| C15:0+C17:0 | 8 | 0.003 | 0.0004 | 2.4 | 0.059 | 0.695 |
| C18:1ω7 | 8 | 0.008 | 0.001 | 7.3 | ***0.001*** | ***0.024*** |
| C20:1+C22:1 | 8 | 0.01 | 0.001 | 21.8 | ***0.001*** | ***0.018*** |
| ARA | 8 | 0.02 | 0.003 | 40.5 | ***0.001*** | 0.595 |
| C24:0 | 8 | 0.000002 | 0.0000003 | 0.4 | 0.9 | 0.8 |
| C18:1ω9 | 8 | 0.009 | 0.0012 | 15.8 | ***0.001*** | ***0.003*** |
| LC_SFA | 8 | 0.01 | 0.001 | 17.8 | ***0.001*** | ***0.006*** |
| C18:2ω6 | 8 | 0.0007 | 0.00008 | 6 | ***0.001*** | 0.066 |
| C20:1ω9 | 8 | 0.0025673 | 0.0003209 | 21 | ***0.001*** | 0.789 |
| PUFA/SFA | 8 | 39.7 | 4.97 | 10 | ***0.001*** | ***0.011*** |

Significant p values are marked in bold and italics.

B

| ***Metachromadora*** | *Praeacanthonchus* | *Theristus* | *Daptonema* | *Oncholaimus* | *Odontophora* | *Enoplus* | *Enoploides* | *Adoncholaimus* |
| --- | --- | --- | --- | --- | --- | --- | --- | --- |
| TFA | 0.351 | ***0.013*** | 0.468 | ***0.012*** | 0.569 | ***0.011*** | ***0.001*** | ***0.011*** |
| PUFA | ***0.005*** | ***0.001*** | ***0.001*** | 0.104 | ***0.013*** | ***0.008*** | ***0.001*** | 0.151 |
| HUFA | ***0.004*** | ***0.001*** | ***0.006*** | ***0.024*** | ***0.01*** | ***0.008*** | ***0.001*** | 0.07 |
| MUFA | ***0.005*** | ***0.003*** | ***0.013*** | ***0.041*** | ***0.032*** | ***0.025*** | ***0.003*** | 0.137 |
| SFA | 0.927 | ***0.001*** | ***0.006*** | ***0.056*** | ***0.015*** | ***0.009*** | ***0.039*** | 0.236 |
| ω3PUFA | ***0.004*** | ***0.001*** | ***0.002*** | 0.912 | ***0.007*** | ***0.019*** | ***0.002*** | 0.989 |
| ω6PUFA | 0.805 | ***0.004*** | ***0.038*** | ***0.006*** | ***0.008*** | ***0.008*** | 0.265 | ***0.008*** |
| C18PUFA | ***0.019*** | ***0.001*** | ***0.001*** | ***0.011*** | ***0.026*** | ***0.007*** | ***0.006*** | ***0.053*** |
| EPA | 0.06 | 0.303 | 0.466 | ***0.002*** | ***0.007*** | ***0.013*** | ***0.003*** | ***0.03*** |
| DHA | ***0.002*** | ***0.001*** | ***0.003*** | ***0.013*** | ***0.013*** | ***0.008*** | ***0.001*** | ***0.031*** |
| EPA/DHA | ***0.001*** | ***0.002*** | ***0.001*** | ***0.015*** | ***0.011*** | ***0.009*** | ***0.001*** | ***0.008*** |
| C16:1ω7 | ***0.004*** | ***0.002*** | ***0.003*** | ***0.007*** | ***0.009*** | ***0.011*** | ***0.002*** | 0.057 |
| C16:1ω7/C16:0 | ***0.004*** | ***0.002*** | ***0.003*** | ***0.009*** | ***0.01*** | ***0.007*** | ***0.002*** | ***0.03*** |
| C14:0+C16:0+C18:0 | 0.79 | ***0.003*** | ***0.009*** | ***0.008*** | ***0.003*** | 0.053 | 0.486 | ***0.008*** |
| C15:0+C17:0 | 0.526 | ***0.004*** | ***0.023*** | 0.252 | 0.086 | 0.481 | 0.359 | 0.235 |
| C18:1ω7 | ***0.014*** | ***0.011*** | 0.403 | 0.228 | ***0.007*** | 0.812 | ***0.001*** | 0.608 |
| C20:1+C22:1 | 0.651 | ***0.001*** | ***0.015*** | ***0.042*** | ***0.012*** | 0.877 | ***0.004*** | 0.48 |
| ARA | 0.594 | ***0.001*** | ***0.001*** | ***0.001*** | ***0.001*** | ***0.001*** | ***0.012*** | ***0.001*** |
| C18_1ω9 | 0.462 | ***0.001*** | ***0.001*** | ***0.009*** | ***0.001*** | ***0.002*** | 0.587 | ***0.001*** |
| LC_SFA | ***0.003*** | ***0.004*** | ***0.002*** | ***0.001*** | 0.067 | ***0.011*** | ***0.005*** | ***0.012*** |
| C18:2ω6 | 0.087 | ***0.001*** | ***0.002*** | ***0.004*** | 0.072 | ***0.029*** | ***0.017*** | ***0.044*** |
| C20:1ω9 | 0.774 | 0.085 | 0.102 | ***0.001*** | ***0.011*** | ***0.01*** | ***0.001*** | 0.067 |
| PUFA/SFA | ***0.022*** | ***0.006*** | ***0.005*** | 0.685 | ***0.004*** | ***0.01*** | ***0.001*** | 0.295 |
| *Praeacanthonchus* |  | *Theristus* | *Daptonema* | *Oncholaimus* | *Odontophora* | *Enoplus* | *Enoploides* | *Adoncholaimus* |
| TFA |  | 0.067 | 0.671 | ***0.004*** | 0.359 | ***0.004*** | ***0.001*** | ***0.008*** |
| PUFA |  | ***0.011*** | ***0.035*** | 0.131 | 0.282 | 0.354 | 0.13 | 0.117 |
| HUFA |  | ***0.004*** | ***0.013*** | 0.431 | 0.174 | 0.331 | 0.13 | 0.168 |
| MUFA |  | 0.854 | 0.784 | 0.357 | 0.694 | 0.988 | 0.252 | 0.154 |
| SFA |  | ***0.001*** | ***0.002*** | 0.368 | ***0.042*** | 0.199 | 0.53 | 0.621 |
| ω3PUFA |  | ***0.041*** | ***0.068*** | ***0.03*** | 0.869 | 0.682 | 0.316 | ***0.029*** |
| ω6PUFA |  | ***0.051*** | 0.196 | ***0.005*** | ***0.005*** | ***0.008*** | 0.555 | ***0.007*** |
| C18PUFA |  | ***0.005*** | ***0.01*** | 0.133 | 0.438 | 0.282 | ***0.053*** | 0.796 |
| EPA |  | 0.176 | 0.06 | ***0.004*** | ***0.004*** | ***0.008*** | ***0.013*** | ***0.017*** |
| DHA |  | ***0.001*** | ***0.001*** | 0.294 | ***0.012*** | 0.438 | ***0.001*** | 0.109 |
| EPA/DHA |  | ***0.003*** | ***0.003*** | 0.097 | ***0.005*** | ***0.031*** | ***0.008*** | 0.687 |
| C16:1ω7 |  | 0.097 | 0.127 | 0.955 | 0.183 | 0.368 | ***0.14*** | ***0.2*** |
| C16:1ω7/C16:0 |  | 0.273 | 0.387 | 0.454 | 0.937 | 0.502 | 0.168 | 0.381 |
| C14:0+C16:0+C18:0 |  | ***0.036*** | ***0.019*** | ***0.031*** | ***0.019*** | 0.663 | 0.628 | 0.084 |
| C15:0+C17:0 |  | ***0.021*** | 0.102 | 0.8 | 0.245 | 0.988 | 0.817 | 0.803 |
| C18:1ω7 |  | 0.474 | 0.069 | ***0.014*** | 0.534 | 0.057 | ***0.002*** | 0.123 |
| C20:1+C22:1 |  | ***0.001*** | ***0.01*** | ***0.019*** | ***0.005*** | 0.845 | ***0.001*** | 0.381 |
| ARA |  | ***0.001*** | ***0.002*** | ***0.001*** | ***0.001*** | ***0.001*** | ***0.039*** | ***0.001*** |
| C18:1ω9 |  | ***0.001*** | ***0.001*** | ***0.002*** | ***0.001*** | ***0.005*** | 0.978 | ***0.001*** |
| LC_SFA |  | ***0.001*** | ***0.003*** | ***0.004*** | 0.251 | ***0.005*** | ***0.001*** | ***0.001*** |
| C18:2ω6 |  | ***0.037*** | 0.051 | 0.266 | 0.945 | 0.674 | 0.082 | 0.993 |
| C20:1ω9 |  | 0.986 | 0.409 | ***0.005*** | ***0.015*** | ***0.004*** | ***0.011*** | 0.911 |
| PUFA/SFA |  | ***0.001*** | ***0.004*** | 0.25 | 0.072 | 0.297 | 0.425 | 0.297 |
| ***Theristus*** |  |  | *Daptonema* | *Oncholaimus* | *Odontophora* | *Enoplus* | *Enoploides* | *Adoncholaimus* |
| TFA |  |  | ***0.012*** | ***0.001*** | ***0.011*** | ***0.001*** | ***0.001*** | ***0.001*** |
| PUFA |  |  | 0.954 | ***0.002*** | 0.111 | 0.067 | 0.715 | ***0.002*** |
| HUFA |  |  | 0.865 | ***0.017*** | 0.054 | ***0.014*** | 0.433 | ***0.001*** |
| MUFA |  |  | 0.782 | 0.089 | 0.265 | 0.522 | ***0.002*** | ***0.001*** |
| SFA |  |  | 0.816 | ***0.001*** | 0.21 | 0.055 | ***0.037*** | ***0.002*** |
| ω3PUFA |  |  | 0.764 | ***0.001*** | ***0.009*** | ***0.003*** | 0.409 | ***0.001*** |
| ω6PUFA |  |  | 0.431 | ***0.001*** | ***0.001*** | ***0.002*** | 0.329 | ***0.001*** |
| C18PUFA |  |  | 0.396 | ***0.205*** | ***0.003*** | ***0.014*** | 0.121 | ***0.002*** |
| EPA |  |  | ***0.016*** | ***0.001*** | ***0.001*** | ***0.001*** | ***0.001*** | ***0.001*** |
| DHA |  |  | 0.167 | ***0.001*** | 0.061 | ***0.002*** | 0.219 | ***0.001*** |
| EPA/DHA |  |  | 0.159 | ***0.001*** | ***0.012*** | 0.203 | ***0.003*** | ***0.001*** |
| C16:1ω7 |  |  | 0.215 | ***0.001*** | 0.095 | 0.127 | ***0.007*** | ***0.001*** |
| C16:1ω7/C16:0 |  |  | 0.996 | ***0.856*** | ***0.048*** | 0.681 | 0.055 | ***0.001*** |
| C14:0+C16:0+C18:0 |  |  | 0.566 | ***0.004*** | 0.607 | 0.129 | 0.072 | ***0.005*** |
| C15:0+C17:0 |  |  | 0.891 | ***0.001*** | ***0.001*** | ***0.001*** | ***0.001*** | ***0.001*** |
| C18:1ω7 |  |  | ***0.037*** | ***0.024*** | 0.916 | ***0.023*** | ***0.008*** | ***0.04*** |
| C20:1+C22:1 |  |  | ***0.009*** | ***0.007*** | ***0.048*** | ***0.001*** | 0.321 | ***0.002*** |
| ARA |  |  | 0.567 | ***0.001*** | ***0.001*** | ***0.001*** | 0.205 | ***0.001*** |
| C18:1ω9 |  |  | ***0.015*** | ***0.553*** | ***0.002*** | ***0.001*** | ***0.001*** | 0.268 |
| LC_SFA |  |  | ***0.008*** | ***0.077*** | ***0.008*** | 0.197 | 0.83 | 0.091 |
| C18:2ω6 |  |  | 0.404 | ***0.157*** | ***0.008*** | ***0.011*** | 0.126 | ***0.003*** |
| C20:1ω9 |  |  | ***0.022*** | ***0.001*** | ***0.001*** | ***0.001*** | ***0.001*** | 0.821 |
| PUFA/SFA |  |  | 0.914 | ***0.011*** | 0.184 | 0.092 | 0.156 | ***0.009*** |
| ***Daptonema*** |  |  |  | *Oncholaimus* | *Odontophora* | *Enoplus* | *Enoploides* | *Adoncholaimus* |
| TFA |  |  |  | ***0.001*** | 0.244 | ***0.002*** | ***0.003*** | ***0.001*** |
| PUFA |  |  |  | ***0.005*** | 0.166 | 0.09 | 0.751 | ***0.003*** |
| HUFA |  |  |  | ***0.002*** | 0.116 | ***0.023*** | 0.423 | ***0.001*** |
| MUFA |  |  |  | 0.353 | 0.851 | 0.672 | 0.072 | ***0.05*** |
| SFA |  |  |  | ***0.004*** | 0.131 | ***0.022*** | ***0.027*** | ***0.002*** |
| ω3PUFA |  |  |  | ***0.001*** | ***0.034*** | ***0.014*** | 0.486 | ***0.001*** |
| ω6PUFA |  |  |  | ***0.002*** | ***0.002*** | ***0.002*** | 0.651 | ***0.003*** |
| C18PUFA |  |  |  | 0.085 | ***0.009*** | ***0.019*** | 0.533 | ***0.001*** |
| EPA |  |  |  | ***0.013*** | ***0.005*** | ***0.026*** | ***0.025*** | 0.08 |
| DHA |  |  |  | ***0.005*** | 0.085 | ***0.011*** | 0.927 | ***0.005*** |
| EPA/DHA |  |  |  | ***0.003*** | 0.772 | 0.096 | 0.317 | ***0.004*** |
| C16:1ω7 |  |  |  | ***0.014*** | 0.963 | 0.163 | 0.225 | ***0.002*** |
| C16:1ω7/C16:0 |  |  |  | 0.894 | 0.133 | 0.767 | 0.144 | ***0.017*** |
| C14:0+C16:0+C18:0 |  |  |  | ***0.003*** | 0.974 | ***0.046*** | ***0.028*** | ***0.002*** |
| C15:0+C17:0 |  |  |  | ***0.001*** | ***0.008*** | ***0.001*** | ***0.001*** | ***0.001*** |
| C18:1ω7 |  |  |  | 0.228 | ***0.003*** | 0.264 | ***0.001*** | 0.773 |
| C20:1+C22:1 |  |  |  | 0.989 | ***0.002*** | ***0.001*** | ***0.002*** | ***0.001*** |
| ARA |  |  |  | ***0.002*** | ***0.001*** | ***0.001*** | 0.281 | ***0.002*** |
| C18:1ω9 |  |  |  | 0.371 | ***0.004*** | 0.18 | ***0.005*** | 0.099 |
| LC_SFA |  |  |  | ***0.002*** | ***0.041*** | 0.063 | ***0.032*** | ***0.07*** |
| C18:2ω6 |  |  |  | 0.103 | ***0.006*** | ***0.011*** | 0.535 | ***0.008*** |
| C20:1ω9 |  |  |  | ***0.001*** | ***0.002*** | ***0.001*** | ***0.001*** | ***0.014*** |
| PUFA/SFA |  |  |  | ***0.007*** | 0.158 | 0.054 | 0.105 | ***0.012*** |
| ***Oncholaimus*** |  |  |  |  | *Odontophora* | *Enoplus* | *Enoploides* | *Adoncholaimus* |
| TFA |  |  |  |  | ***0.001*** | ***0.001*** | 0.159 | ***0.001*** |
| PUFA |  |  |  |  | ***0.011*** | ***0.016*** | ***0.02*** | 0.736 |
| HUFA |  |  |  |  | ***0.016*** | ***0.017*** | ***0.025*** | 0.335 |
| MUFA |  |  |  |  | 0.362 | 0.152 | 0.058 | 0.412 |
| SFA |  |  |  |  | ***0.013*** | ***0.017*** | 0.067 | 0.429 |
| ω3PUFA |  |  |  |  | ***0.004*** | ***0.001*** | ***0.006*** | 0.773 |
| ω6PUFA |  |  |  |  | ***0.092*** | ***0.076*** | ***0.015*** | 0.052 |
| C18PUFA |  |  |  |  | ***0.003*** | ***0.004*** | ***0.002*** | ***0.001*** |
| EPA |  |  |  |  | 0.595 | 0.225 | 0.408 | ***0.045*** |
| DHA |  |  |  |  | ***0.001*** | ***0.004*** | ***0.006*** | 0.057 |
| EPA/DHA |  |  |  |  | ***0.001*** | ***0.009*** | ***0.003*** | ***0.001*** |
| C16:1ω7 |  |  |  |  | ***0.01*** | ***0.02*** | ***0.015*** | ***0.008*** |
| C16:1ω7/C16:0 |  |  |  |  | 0.064 | 0.763 | 0.08 | ***0.004*** |
| C14:0+C16:0+C18:0 |  |  |  |  | ***0.001*** | ***0.008*** | 0.053 | 0.293 |
| C15:0+C17:0 |  |  |  |  | ***0.005*** | ***0.03*** | 0.537 | 0.819 |
| C18:1ω7 |  |  |  |  | 0.109 | 0.386 | 0.069 | 0.328 |
| C20:1+C22:1 |  |  |  |  | ***0.038*** | ***0.003*** | ***0.017*** | ***0.008*** |
| ARA |  |  |  |  | ***0.049*** | ***0.02*** | ***0.009*** | ***0.007*** |
| C18:1ω9 |  |  |  |  | 0.954 | 0.328 | 0.191 | 0.63 |
| LC_SFA |  |  |  |  | ***0.054*** | ***0.001*** | ***0.001*** | ***0.001*** |
| C18:2ω6 |  |  |  |  | ***0.003*** | ***0.003*** | ***0.001*** | ***0.004*** |
| C20:1ω9 |  |  |  |  | ***0.001*** | ***0.001*** | ***0.002*** | ***0.002*** |
| PUFA/SFA |  |  |  |  | ***0.011*** | ***0.005*** | ***0.014*** | 0.742 |
| ***Odontophora*** |  |  |  |  |  | *Enoplus* | *Enoploides* | *Adoncholaimus* |
| TFA |  |  |  |  |  | ***0.001*** | ***0.013*** | ***0.001*** |
| PUFA |  |  |  |  |  | 0.635 | 0.256 | ***0.004*** |
| HUFA |  |  |  |  |  | 0.261 | 0.345 | ***0.001*** |
| MUFA |  |  |  |  |  | 0.067 | ***0.011*** | ***0.003*** |
| SFA |  |  |  |  |  | 0.271 | 0.146 | ***0.008*** |
| ω3PUFA |  |  |  |  |  | 0.201 | 0.158 | ***0.001*** |
| ω6PUFA |  |  |  |  |  | 0.891 | ***0.015*** | 0.435 |
| C18PUFA |  |  |  |  |  | ***0.034*** | ***0.003*** | ***0.03*** |
| EPA |  |  |  |  |  | ***0.031*** | 0.467 | ***0.007*** |
| DHA |  |  |  |  |  | ***0.004*** | ***0.031*** | ***0.001*** |
| EPA/DHA |  |  |  |  |  | ***0.008*** | 0.052 | ***0.001*** |
| C16:1ω7 |  |  |  |  |  | ***0.016*** | 0.089 | ***0.001*** |
| C16:1ω7/C16:0 |  |  |  |  |  | ***0.042*** | ***0.015*** | ***0.003*** |
| C14:0+C16:0+C18:0 |  |  |  |  |  | ***0.005*** | ***0.001*** | ***0.001*** |
| C15:0+C17:0 |  |  |  |  |  | ***0.001*** | ***0.002*** | ***0.001*** |
| C18:1ω7 |  |  |  |  |  | ***0.007*** | ***0.006*** | ***0.007*** |
| C20:1+C22:1 |  |  |  |  |  | ***0.001*** | 0.12 | ***0.002*** |
| ARA |  |  |  |  |  | 0.292 | ***0.009*** | ***0.035*** |
| C18:1ω9 |  |  |  |  |  | ***0.002*** | ***0.001*** | ***0.003*** |
| LC_SFA |  |  |  |  |  | 0.06 | 0.148 | 0.083 |
| C18:2ω6 |  |  |  |  |  | ***0.033*** | ***0.002*** | 0.683 |
| C20:1ω9 |  |  |  |  |  | ***0.019*** | 0.011 | ***0.001*** |
| PUFA/SFA |  |  |  |  |  | 0.313 | 0.38 | ***0.015*** |
| ***Enoplus*** |  |  |  |  |  |  | *Enoploides* | *Adoncholaimus* |
| TFA |  |  |  |  |  |  | ***0.005*** | 0.088 |
| PUFA |  |  |  |  |  |  | 0.075 | ***0.001*** |
| HUFA |  |  |  |  |  |  | ***0.041*** | ***0.001*** |
| MUFA |  |  |  |  |  |  | ***0.003*** | ***0.001*** |
| SFA |  |  |  |  |  |  | 0.167 | ***0.009*** |
| ω3PUFA |  |  |  |  |  |  | ***0.023*** | ***0.001*** |
| ω6PUFA |  |  |  |  |  |  | ***0.011*** | 0.403 |
| C18PUFA |  |  |  |  |  |  | ***0.001*** | ***0.001*** |
| EPA |  |  |  |  |  |  | 0.06 | ***0.042*** |
| DHA |  |  |  |  |  |  | ***0.01*** | ***0.001*** |
| EPA/DHA |  |  |  |  |  |  | ***0.014*** | ***0.002*** |
| C16:1ω7 |  |  |  |  |  |  | ***0.013*** | ***0.001*** |
| C16:1ω7/C16:0 |  |  |  |  |  |  | ***0.042*** | ***0.002*** |
| C14:0+C16:0+C18:0 |  |  |  |  |  |  | 0.081 | ***0.005*** |
| C15:0+C17:0 |  |  |  |  |  |  | 0.059 | ***0.004*** |
| C18:1ω7 |  |  |  |  |  |  | ***0.002*** | 0.24 |
| C20:1+C22:1 |  |  |  |  |  |  | ***0.001*** | ***0.007*** |
| ARA |  |  |  |  |  |  | ***0.01*** | 0.074 |
| C18:1ω9 |  |  |  |  |  |  | ***0.005*** | ***0.009*** |
| LC_SFA |  |  |  |  |  |  | ***0.006*** | 0.053 |
| C18:2ω6 |  |  |  |  |  |  | ***0.002*** | ***0.045*** |
| C20:1ω9 |  |  |  |  |  |  | 0.407 | ***0.001*** |
| PUFA/SFA |  |  |  |  |  |  | 0.876 | ***0.001*** |
| ***Enoploides*** |  |  |  |  |  |  |  | *Adoncholaimus* |
| TFA |  |  |  |  |  |  |  | ***0.002*** |
| PUFA |  |  |  |  |  |  |  | ***0.003*** |
| HUFA |  |  |  |  |  |  |  | ***0.003*** |
| MUFA |  |  |  |  |  |  |  | ***0.002*** |
| SFA |  |  |  |  |  |  |  | ***0.032*** |
| ω3PUFA |  |  |  |  |  |  |  | ***0.003*** |
| ω6PUFA |  |  |  |  |  |  |  | ***0.028*** |
| C18PUFA |  |  |  |  |  |  |  | ***0.002*** |
| EPA |  |  |  |  |  |  |  | ***0.011*** |
| DHA |  |  |  |  |  |  |  | ***0.003*** |
| EPA/DHA |  |  |  |  |  |  |  | ***0.001*** |
| C16:1ω7 |  |  |  |  |  |  |  | ***0.002*** |
| C16:1ω7/C16:0 |  |  |  |  |  |  |  | ***0.006*** |
| C14:0+C16:0+C18:0 |  |  |  |  |  |  |  | ***0.032*** |
| C15:0+C17:0 |  |  |  |  |  |  |  | 0.184 |
| C18:1ω7 |  |  |  |  |  |  |  | ***0.001*** |
| C20:1+C22:1 |  |  |  |  |  |  |  | ***0.001*** |
| ARA |  |  |  |  |  |  |  | ***0.024*** |
| C18:1ω9 |  |  |  |  |  |  |  | ***0.001*** |
| LC_SFA |  |  |  |  |  |  |  | ***0.004*** |
| C18:2ω6 |  |  |  |  |  |  |  | ***0.001*** |
| C20:1ω9 |  |  |  |  |  |  |  | ***0.002*** |
| PUFA/SFA |  |  |  |  |  |  |  | ***0.005*** |

Significant p values are marked in bold and italics.

Table S3. **Results of similarity percentage analysis.**

Dissimilarity in the FA profiles between pairs of nematode species, and the main fatty acids responsible for these dissimilarities (listed here up to a cumulative contribution of ca 50 % of the total dissimilarity) as detected using SIMPER (similarity percentage analysis).

| Compare groups | | AV Diss | FA | Average abundance (Prop) | | Av.Diss±SD | Contrib% |
| --- | --- | --- | --- | --- | --- | --- | --- |
| Species 1 | Species 2 | % |  | Species 1 | Species 2 |  |  |
| *Metachromadora* | *Praeacanthonchus* | 24 | C16:1ω7 | 0.19 | 0.08 | 5.4±2.1 | 22.8 |
|  |  |  | DHA | 0.07 | 0.14 | 3.5±2 | 14.7 |
|  |  |  | EPA | 0.26 | 0.3 | 3±1.5 | 12.7 |
| *Metachromadora* | *Theristus* | 34 | DHA | 0.07 | 0.25 | 8.6±5.8 | 25 |
|  |  |  | C16:1ω7 | 0.19 | 0.05 | 6.9±3.5 | 20 |
|  |  |  | C16:0 | 0.13 | 0.09 | 1.9±1.6 | 5.5 |
| *Metachromadora* | *Daptonema* | 36 | DHA | 0.07 | 0.29 | 10.5±4.1 | 29.3 |
|  |  |  | C16:1ω7 | 0.19 | 0.04 | 7.3±3.5 | 20.2 |
| *Metachromadora* | *Oncholaimus* | 32 | C16:1ω7 | 0.19 | 0.08 | 5.2±2.5 | 16.4 |
|  |  |  | EPA | 0.26 | 0.18 | 3.6±2.6 | 11.5 |
|  |  |  | ARA | 0 | 0.07 | 3.6±5.9 | 11.3 |
|  |  |  | C18:0 | 0.06 | 0.1 | 2.3±2.7 | 7.4 |
|  |  |  | DHA | 0.07 | 0.12 | 2.1±2.5 | 6.7 |
| *Metachromadora* | *Odontophora* | 43 | C16:1ω7 | 0.19 | 0.04 | 7.3±3.6 | 16.7 |
|  |  |  | DHA | 0.07 | 0.21 | 6.8±7.6 | 15.7 |
|  |  |  | C22:5ω3 | 0.01 | 0.09 | 4±12.5 | 9.1 |
|  |  |  | EPA | 0.26 | 0.18 | 3.9±3 | 9 |
| *Metachromadora* | *Enoplus* | 35 | C16:1ω7 | 0.19 | 0.05 | 6.6±3.3 | 18.9 |
|  |  |  | DHA | 0.07 | 0.16 | 4.3±4.8 | 12.4 |
|  |  |  | C22:5ω3 | 0.01 | 0.1 | 4.1±11.5 | 11.9 |
|  |  |  | EPA | 0.26 | 0.2 | 3±2.3 | 8.5 |
| *Metachromadora* | *Enoploides* | 49 | DHA | 0.07 | 0.28 | 10.3±6.9 | 21.1 |
|  |  |  | C16:1ω7 | 0.19 | 0.02 | 8.1±3.9 | 16.7 |
|  |  |  | EPA | 0.26 | 0.17 | 4.3±3.1 | 8.8 |
|  |  |  | C18:0 | 0.06 | 0.13 | 3.8±14.4 | 7.7 |
| *Metachromadora* | *Adoncholaimus* | 22 | C16:1ω7 | 0.19 | 0.13 | 3.3±3.4 | 15 |
|  |  |  | EPA | 0.26 | 0.21 | 2.2±1.7 | 9.9 |
|  |  |  | ARA | 0 | 0.05 | 2.2±4.6 | 9.8 |
|  |  |  | C22:5ω3 | 0.01 | 0.05 | 1.7±6.8 | 7.6 |
|  |  |  | C18:0 | 0.06 | 0.09 | 1.6±2.9 | 7.4 |
| *Praeacanthonchus* | *Theristus* | 26 | DHA | 0.14 | 0.25 | 5.2±2.4 | 20 |
|  |  |  | EPA | 0.3 | 0.27 | 2.6±1.7 | 10.1 |
|  |  |  | C16:1ω7 | 0.08 | 0.05 | 2.1±0.9 | 8 |
|  |  |  | C16:0 | 0.11 | 0.09 | 1.6±1.4 | 6.2 |
|  |  |  | C22:1ω9 | 0.02 | 0.05 | 1.6±2.8 | 5.9 |
| *Praeacanthonchus* | *Daptonema* | 29 | DHA | 0.14 | 0.29 | 7.2±2.4 | 24.8 |
|  |  |  | EPA | 0.3 | 0.24 | 3.3±1.5 | 11.4 |
|  |  |  | C16:1ω7 | 0.08 | 0.04 | 2.4±1 | 8.3 |
|  |  |  | C16:0 | 0.11 | 0.07 | 2.3±2.6 | 7.8 |
| *Praeacanthonchus* | *Oncholaimus* | 30 | EPA | 0.3 | 0.18 | 5.9±2.2 | 19.5 |
|  |  |  | ARA | 0.01 | 0.07 | 3.4±5.2 | 11.4 |
|  |  |  | DHA | 0.14 | 0.12 | 2±2.3 | 6.7 |
|  |  |  | C16:1ω7 | 0.08 | 0.08 | 2±1.1 | 6.5 |
|  |  |  | C18:0 | 0.07 | 0.1 | 1.8±1.7 | 5.9 |
| *Praeacanthonchus* | *Odontophora* | 34 | EPA | 0.3 | 0.18 | 6.1±2.4 | 18 |
|  |  |  | DHA | 0.14 | 0.21 | 3.5±1.9 | 10.2 |
|  |  |  | C22:5ω3 | 0.03 | 0.09 | 3±5.9 | 8.9 |
|  |  |  | C16:0 | 0.11 | 0.05 | 2.9±3 | 8.5 |
|  |  |  | ARA | 0.01 | 0.06 | 2.5±4.4 | 7.4 |
| *Praeacanthonchus* | *Enoplus* | 28 | EPA | 0.3 | 0.2 | 5.2±2 | 18.4 |
|  |  |  | C22:5ω3 | 0.03 | 0.1 | 3.2±5.9 | 11.2 |
|  |  |  | ARA | 0.01 | 0.05 | 2.3±4.2 | 8.2 |
|  |  |  | C16:1ω7 | 0.08 | 0.05 | 1.9±0.9 | 6.8 |
|  |  |  | C16:0 | 0.11 | 0.09 | 1.4±1.9 | 5.1 |
| *Praeacanthonchus* | *Enoploides* | 39 | DHA | 0.14 | 0.28 | 6.9±3.2 | 17.7 |
|  |  |  | EPA | 0.3 | 0.17 | 6.5±2.5 | 16.7 |
|  |  |  | C18:0 | 0.07 | 0.13 | 3.2±5 | 8.3 |
|  |  |  | C16:1ω7 | 0.08 | 0.02 | 3.1±1.2 | 7.9 |
| *Praeacanthonchus* | *Adoncholaimus* | 25 | EPA | 0.3 | 0.21 | 4.4±1.7 | 17.8 |
|  |  |  | C16:1ω7 | 0.08 | 0.13 | 3±2 | 12.1 |
|  |  |  | DHA | 0.14 | 0.1 | 2.5±2.2 | 9.9 |
|  |  |  | ARA | 0.01 | 0.05 | 2±3.7 | 8.1 |
|  |  |  | C18:1ω9 | 0 | 0.03 | 1.4±3.5 | 5.4 |
| *Theristus* | *Daptonema* | 14 | DHA | 0.25 | 0.29 | 3±2 | 21.9 |
|  |  |  | C22:1ω9 | 0.05 | 0.02 | 1.5±4.5 | 10.8 |
|  |  |  | EPA | 0.27 | 0.24 | 1.4±1.4 | 10 |
|  |  |  | C24:1ω9 | 0 | 0.03 | 1.3±1.1 | 9.9 |
| *Theristus* | *Oncholaimus* | 27 | DHA | 0.25 | 0.12 | 6.5±4.9 | 23.7 |
|  |  |  | EPA | 0.27 | 0.18 | 4.2±6.2 | 15.5 |
|  |  |  | C18:0 | 0.05 | 0.1 | 2.9±2.9 | 10.6 |
| *Theristus* | *Odontophora* | 23 | EPA | 0.27 | 0.18 | 4.5±10.5 | 19.9 |
|  |  |  | C22:1ω9 | 0.05 | 0 | 2.4±7.2 | 10.8 |
|  |  |  | C22:5ω3 | 0.05 | 0.09 | 2.1±7.2 | 9.4 |
|  |  |  | DHA | 0.25 | 0.21 | 1.8±1.4 | 7.9 |
|  |  |  | C16:0 | 0.09 | 0.05 | 1.7±1.5 | 7.7 |
| *Theristus* | *Enoplus* | 23 | DHA | 0.25 | 0.16 | 4.3±3.2 | 18.6 |
|  |  |  | EPA | 0.27 | 0.2 | 3.6±9.2 | 15.5 |
|  |  |  | C22:5ω3 | 0.05 | 0.1 | 2.3±6.8 | 9.9 |
|  |  |  | C22:1ω9 | 0.05 | 0.01 | 2.2±6.8 | 9.3 |
| *Theristus* | *Enoploides* | 24 | EPA | 0.27 | 0.17 | 4.9±8.4 | 20.2 |
|  |  |  | C18:0 | 0.05 | 0.13 | 4.4±7.2 | 18.1 |
|  |  |  | DHA | 0.25 | 0.28 | 2±1.6 | 8.4 |
|  |  |  | C22:5ω3 | 0.05 | 0.08 | 1.7±3 | 7.1 |
| *Theristus* | *Adoncholaimus* | 26 | DHA | 0.25 | 0.1 | 7.3±5.7 | 27.6 |
|  |  |  | C16:1ω7 | 0.05 | 0.13 | 4±12.6 | 15.2 |
|  |  |  | EPA | 0.27 | 0.21 | 2.8±7 | 10.6 |
| *Daptonema* | *Oncholaimus* | 30 | DHA | 0.29 | 0.12 | 8.4±3.3 | 28 |
|  |  |  | C16:0 | 0.07 | 0.14 | 3.6±5.2 | 11.8 |
|  |  |  | EPA | 0.24 | 0.18 | 3±2.4 | 9.8 |
| *Daptonema* | *Odontophora* | 22 | DHA | 0.29 | 0.21 | 3.9±1.7 | 17.4 |
|  |  |  | EPA | 0.24 | 0.18 | 3.2±3 | 14.5 |
|  |  |  | C22:5ω3 | 0.04 | 0.09 | 2.7±4.6 | 12.3 |
|  |  |  | C20:1 | 0.02 | 0.05 | 1.5±19.3 | 6.8 |
| *Daptonema* | *Enoplus* | 23 | DHA | 0.29 | 0.16 | 6.2±2.5 | 27.5 |
|  |  |  | C22:5ω3 | 0.04 | 0.1 | 2.9±4.7 | 12.8 |
|  |  |  | EPA | 0.24 | 0.2 | 2.3±2.1 | 10.1 |
| *Daptonema* | *Enoploides* | 24 | C18:0 | 0.05 | 0.13 | 3.9±6.6 | 16.7 |
|  |  |  | EPA | 0.24 | 0.17 | 3.6±3 | 15.3 |
|  |  |  | C18:1ω7 | 0.08 | 0.03 | 2.4±8.2 | 10 |
|  |  |  | C22:5ω3 | 0.04 | 0.08 | 2.3±3 | 9.9 |
| *Daptonema* | *Adoncholaimus* | 28 | DHA | 0.29 | 0.1 | 9.2±3.7 | 33.4 |
|  |  |  | C16:1ω7 | 0.04 | 0.13 | 4.4±6.7 | 16 |
| *Oncholaimus* | *Odontophora* | 29 | DHA | 0.12 | 0.21 | 4.7±8.9 | 16.3 |
|  |  |  | C16:0 | 0.14 | 0.05 | 4.4±14.3 | 15 |
|  |  |  | C22:5ω3 | 0.05 | 0.09 | 2.1±6.7 | 7.3 |
|  |  |  | C16:1ω7 | 0.08 | 0.04 | 2.1±3.4 | 7.2 |
|  |  |  | C20:1 | 0.01 | 0.05 | 1.9±29.4 | 6.6 |
| *Oncholaimus* | *Enoplus* | 20 | C16:0 | 0.14 | 0.09 | 2.5±5.4 | 12.7 |
|  |  |  | C22:5ω3 | 0.05 | 0.1 | 2.3±6.3 | 11.3 |
|  |  |  | DHA | 0.12 | 0.16 | 2.2±4.1 | 11 |
|  |  |  | C20:1ω9 | 0 | 0.04 | 1.8±11.4 | 8.8 |
|  |  |  | C16:1ω7 | 0.08 | 0.05 | 1.4±2.4 | 7 |
| *Oncholaimus* | *Enoploides* | 32 | DHA | 0.12 | 0.28 | 8.2±6 | 25.2 |
|  |  |  | C16:0 | 0.14 | 0.07 | 3.7±11 | 11.5 |
|  |  |  | C18:1ω7 | 0.1 | 0.03 | 3.4±2.3 | 10.6 |
|  |  |  | C16:1ω7 | 0.08 | 0.02 | 3±4 | 9.1 |
| *Oncholaimus* | *Adoncholaimus* | 15 | C16:1ω7 | 0.08 | 0.13 | 2.3±3.9 | 15.6 |
|  |  |  | EPA | 0.18 | 0.21 | 1.4±2.1 | 9.7 |
|  |  |  | ARA | 0.07 | 0.05 | 1.4±3.3 | 9.4 |
|  |  |  | C18:1ω7 | 0.1 | 0.08 | 1±0.7 | 7.1 |
|  |  |  | C22:1ω9 | 0.03 | 0.01 | 0.9±5.2 | 6.3 |
|  |  |  | C18:1ω9 | 0.04 | 0.03 | 0.9±1.1 | 6.2 |
| *Odontophora* | *Enoplus* | 17 | DHA | 0.21 | 0.16 | 2.5±4.5 | 15.3 |
|  |  |  | C16:0 | 0.05 | 0.09 | 1.8±3.8 | 11 |
|  |  |  | C20:1 | 0.05 | 0.02 | 1.6±22 | 9.4 |
|  |  |  | C20:0 | 0.03 | 0 | 1.5±1.2 | 9.3 |
|  |  |  | C18:1ω7 | 0.06 | 0.08 | 1±4.7 | 5.8 |
| *Odontophora* | *Enoploides* | 23 | DHA | 0.21 | 0.28 | 3.4±2.5 | 14.6 |
|  |  |  | C18:0 | 0.07 | 0.13 | 3.3±18.6 | 14.2 |
|  |  |  | C22:1ω9 | 0 | 0.05 | 2.2±14.7 | 9.2 |
|  |  |  | C20:1 | 0.05 | 0.01 | 1.8±29.6 | 7.8 |
|  |  |  | C18:1ω9 | 0.04 | 0 | 1.8±48.4 | 7.8 |
| *Odontophora* | *Adoncholaimus* | 29 | DHA | 0.21 | 0.1 | 5.5±12.1 | 19.1 |
|  |  |  | C16:1ω7 | 0.04 | 0.13 | 4.4±15.1 | 15.3 |
|  |  |  | C16:0 | 0.05 | 0.14 | 4.1±17.7 | 14.1 |
|  |  |  | C22:5ω3 | 0.09 | 0.05 | 2.3±9.6 | 7.9 |
| *Enoplus* | *Enoploides* | 23 | DHA | 0.16 | 0.28 | 6±4.4 | 26.1 |
|  |  |  | C18:0 | 0.08 | 0.13 | 2.7±4.8 | 11.7 |
|  |  |  | C18:1ω7 | 0.08 | 0.03 | 2.5±10.1 | 10.9 |
|  |  |  | C22:1ω9 | 0.01 | 0.05 | 1.9±25.4 | 8.3 |
| *Enoplus* | *Adoncholaimus* | 18 | C16:1ω7 | 0.05 | 0.13 | 3.7±17.7 | 20.5 |
|  |  |  | DHA | 0.16 | 0.1 | 3±6.4 | 16.6 |
|  |  |  | C22:5ω3 | 0.1 | 0.05 | 2.4±8.3 | 13.5 |
| *Enoploides* | *Adoncholaimus* | 35 | DHA | 0.28 | 0.1 | 8.9±6.8 | 25.2 |
|  |  |  | C16:1ω7 | 0.02 | 0.13 | 5.3±10.7 | 14.8 |
|  |  |  | C16:0 | 0.07 | 0.14 | 3.4±12.8 | 9.7 |

Table S4. **Result of PERMANOVA tests on the FA composition of nematodes.**

Results are shown of a one-way PERMANOVA with factor species and of a two-way PERMANOVA with factors species and station. The former included all nematode samples, while the latter only included information on three species that were present at both st1 and st16.

| PERMANOVA | Source | df | SS | MS | Pseudo-F/t | P |
| --- | --- | --- | --- | --- | --- | --- |
| main test | Species | 8 | 12676 | 1585 | 16 | ***0.001*** |
| pairwise test | *Metachromadora, Praeacanthonchus* |  |  |  | 2.9 | ***0.007*** |
|  | *Metachromadora, Theristus* |  |  |  | 7.7 | ***0.002*** |
|  | *Metachromadora, Oncholaimus* |  |  |  | 5.1 | ***0.013*** |
|  | *Metachromadora, Odontophora* |  |  |  | 7.6 | ***0.008*** |
|  | *Metachromadora, Daptonema* |  |  |  | 6.2 | ***0.005*** |
|  | *Metachromadora, Enoplus* |  |  |  | 6.1 | ***0.013*** |
|  | *Metachromadora, Enoploides* |  |  |  | 6.9 | ***0.001*** |
|  | *Metachromadora, Adoncholaimus* |  |  |  | 3.7 | ***0.01*** |
|  | *Praeacanthonchus, Theristus* |  |  |  | 3.4 | ***0.001*** |
|  | *Praeacanthonchus, Oncholaimus* |  |  |  | 2.8 | ***0.009*** |
|  | *Praeacanthonchus, Odontophora* |  |  |  | 3.4 | ***0.006*** |
|  | *Praeacanthochus, Daptonema* |  |  |  | 3.0 | ***0.001*** |
|  | *Praeacanthochus, Enoplus* |  |  |  | 2.7 | ***0.005*** |
|  | *Praeacanthochus, Enoploides* |  |  |  | 3.2 | ***0.001*** |
|  | *Praeacanthochus, Adoncholaimus* |  |  |  | 2.3 | ***0.014*** |
|  | *Theristus, Oncholaimus* |  |  |  | 6.2 | ***0.001*** |
|  | *Theristus, Odontophora* |  |  |  | 5.9 | ***0.001*** |
|  | *Theristus, Daptonema* |  |  |  | 2.4 | ***0.008*** |
|  | *Theristus, Enoplus* |  |  |  | 6.3 | ***0.001*** |
|  | *Theristus, Enoploides* |  |  |  | 5.1 | ***0.002*** |
|  | *Theristus, Adoncholaimus* |  |  |  | 7.3 | ***0.001*** |
|  | *Oncholaimus, Odontophora* |  |  |  | 6.2 | ***0.002*** |
|  | *Oncholaimus, Daptonema* |  |  |  | 4.9 | ***0.003*** |
|  | *Oncholaimus, Enoplus* |  |  |  | 4.4 | ***0.011*** |
|  | *Oncholaimus, Enoploides* |  |  |  | 5.4 | ***0.007*** |
|  | *Oncholaimus, Adoncholaimus* |  |  |  | 3.2 | ***0.007*** |
|  | *Odontophora, Daptonema* |  |  |  | 4.0 | ***0.003*** |
|  | *Odontophora, Enoplus* |  |  |  | 5.8 | ***0.002*** |
|  | *Odontophora, Enoploides* |  |  |  | 5.7 | ***0.006*** |
|  | *Odontophora, Adoncholaimus* |  |  |  | 10.5 | ***0.001*** |
|  | *Daptonema, Enoplus* |  |  |  | 4.3 | ***0.003*** |
|  | *Daptonema, Enoploides* |  |  |  | 3.3 | ***0.013*** |
|  | *Daptonema, Adoncholaimus* |  |  |  | 5.3 | ***0.003*** |
|  | *Enoplus, Enoploides* |  |  |  | 6.2 | ***0.004*** |
|  | *Enoplus, Adoncholaimus* |  |  |  | 7.8 | ***0.003*** |
|  | *Enoploides, Adoncholaimus* |  |  |  | 10.0 | ***0.001*** |
| main test | Species | 2 | 3990 | 1995 | 14 | ***0.001*** |
|  | Station | 1 | 176 | 176 | 1 | 0.274 |
|  | SpeciesxStation | 2 | 307 | 154 | 1 | 0.35 |
| pairwise test | *Metachromadora, Praeacanthonchus* |  |  |  | 1.9 | ***0.035*** |
|  | *Metachromadora, Theristus* |  |  |  | 8.6 | ***0.001*** |
|  | *Praeacanthonchus, Theristus* |  |  |  | 3.0 | ***0.001*** |

Significant p values are marked in bold and italics.

Table S5. **Concentration of six fatty acid markers in three nematode species and related PERMANOVA tests.**

Two-way PERMANOVA results (A) based on a Euclidean distance matrix of the relative abundances of individual fatty acid markers (B) in three nematode species (*Metachromadora remanei*, *Praeacanthonchus punctatus* and *Theristus* *acer*) at two stations (st1 and st16).

A

| Markers | Factor | Source/Groups | df | SS | MS | Pseudo-F/t | P(MC) | p-PERMDISP |
| --- | --- | --- | --- | --- | --- | --- | --- | --- |
| C16:1ω7 |  | Sp | 2 | 0.059 | 0.029 | 14.4 | ***0.002*** | 0.219 |
|  |  | St | 1 | 0.002 | 0.002 | 0.8 | 0.407 | 0.253 |
|  |  | SpxSt** | 2 | 0.001 | 0.000 | 0.2 | 0.779 |  |
|  |  | *Metachromadora, Praeacanthonchus* |  |  |  | 2.5 | ***0.033*** |  |
|  |  | *Metachromadora, Theristus* |  |  |  | 8.0 | ***0.001*** |  |
|  |  | *Praeacanthonchus, Theristus* |  |  |  | 1.9 | 0.091 |  |
| EPA |  | Sp | 2 | 0.001 | 0.000 | 0.2 | 0.832 | 0.177 |
|  |  | St | 1 | 0.002 | 0.002 | 1.3 | 0.259 | 0.92 |
|  |  | SpxSt | 2 | 0.003 | 0.001 | 1.1 | 0.391 |  |
| EPA/DHA |  | Sp | 2 | 18.4 | 9.2 | 74.6 | ***0.001*** | ***0.042*** |
|  |  | St | 1 | 1.9 | 1.9 | 15.2 | ***0.004*** | 0.693 |
|  |  | SpxSt | 2 | 1.1 | 0.5 | 4.5 | ***0.032*** |  |
|  | *Metachromadora* | st1 vs st16 |  |  |  | 1.9 | 0.275 |  |
|  | *Praeacanthonchus* | st1 vs st16 |  |  |  | 3.3 | ***0.034*** |  |
|  | *Theristus* | st1 vs st16 |  |  |  | 11.1 | ***0.002*** |  |
|  | st1 | *Metachromadora, Praeacanthonchus* |  |  |  | 5.0 | ***0.021*** |  |
|  | st1 | *Metachromadora, Theristus* |  |  |  | 8.1 | ***0.002*** |  |
|  | st1 | *Praeacanthonchus, Theristus* |  |  |  | 11.3 | ***0.004*** |  |
|  | st16 | *Metachromadora, Praeacanthonchus* |  |  |  | 2.7 | ***0.025*** |  |
|  | st16 | *Metachromadora, Theristus* |  |  |  | 16.5 | ***0.001*** |  |
|  | st16 | *Praeacanthonchus, Theristus* |  |  |  | 4.9 | ***0.004*** |  |
| DHA |  | Sp | 2 | 0.095 | 0.048 | 103.3 | ***0.001*** | ***0.017*** |
|  |  | St | 1 | 0.006 | 0.006 | 14.0 | ***0.003*** | 0.38 |
|  |  | SpxSt | 2 | 0.002 | 0.001 | 2.0 | 0.179 |  |
|  |  | *Metachromadora, Praeacanthonchus* |  |  |  | 4.1 | ***0.006*** |  |
|  |  | *Metachromadora, Theristus* |  |  |  | 22.6 | ***0.001*** |  |
|  |  | *Praeacanthonchus, Theristus* |  |  |  | 7.9 | ***0.001*** |  |
|  |  | st1 vs st16 |  |  |  | 3.7 | ***0.003*** |  |
| C15:0+C17:0 |  | Sp | 2 | 0.002 | 0.001 | 3.8 | 0.052 | 0.188 |
|  |  | St | 1 | 0.000 | 0.000 | 0.0 | 0.906 | 0.712 |
|  |  | SpxSt | 2 | 0.001 | 0.000 | 1.5 | 0.273 |  |
| C18:1ω7 |  | Sp | 2 | 0.001 | 0.001 | 5.3 | ***0.022*** | 0.479 |
|  |  | St | 2 | 0.000 | 0.000 | 1.3 | 0.269 | 0.724 |
|  |  | SpxSt** | 2 | 0.000 | 0.000 | 0.1 | 0.91 |  |
|  |  | *Metachromadora, Praeacanthonchus* |  |  |  | 2.3 | 0.06 |  |
|  |  | *Metachromadora, Theristus* |  |  |  | 3.7 | ***0.006*** |  |
|  |  | *Praeacanthonchus, Theristus* |  |  |  | 0.4 | 0.675 |  |

Significant p values are marked in bold and italics.

B

|  |  | C16:1ω7 | EPA | EPA/DHA | DHA | C15:0+C17:0 | C18:1ω7 |
| --- | --- | --- | --- | --- | --- | --- | --- |
| *Metachromadora* | st1 | 0.163592 | 0.25614 | 3.312311 | 0.080111 | 0.039441 | 0.078018 |
|  | st16 | 0.201582 | 0.254604 | 3.647944 | 0.069994 | 0.026169 | 0.082755 |
| *Praeacanthonchus* | st1 | 0.09811 | 0.238184 | 1.462478 | 0.163041 | 0.017153 | 0.061451 |
|  | st16 | 0.108687 | 0.292128 | 2.824593 | 0.10826 | 0.03688 | 0.066434 |
| *Theristus* | st1 | 0.044532 | 0.265362 | 0.980465 | 0.270653 | 0.008603 | 0.055935 |
|  | st16 | 0.05142 | 0.269549 | 1.214655 | 0.221955 | 0.008643 | 0.065388 |
